# Systems-Scale Analysis Reveals Pathways Involved in Cellular Response to Methamphetamine

**DOI:** 10.1371/journal.pone.0018215

**Published:** 2011-04-20

**Authors:** Lijie Sun, Hong-Mei Li, Manfredo J. Seufferheld, Kent R. Walters, Venu M. Margam, Amber Jannasch, Naomi Diaz, Catherine P. Riley, Weilin Sun, Yueh-Feng Li, William M. Muir, Jun Xie, Jing Wu, Fan Zhang, Jake Y. Chen, Eric L. Barker, Jiri Adamec, Barry R. Pittendrigh

**Affiliations:** 1 Department of Entomology, University of Illinois at Urbana-Champaign, Urbana, Illinois, United States of America; 2 Synthetic Biology & Bioenergy, J. Craig Venter Institute, San Diego, California, United States of America; 3 Department of Entomology, Purdue University, West Lafayette, Indiana, United States of America; 4 Department of Crop Sciences, University of Illinois at Urbana-Champaign, Urbana, Illinois, United States of America; 5 Metabolomics Profiling Facility at Bindley Bioscience Center, Purdue University, West Lafayette, Indiana, United States of America; 6 Chung Hwa College of Medical Technology, Jen-Te Hsiang, Tainan, Taiwan; 7 Department of Animal Sciences, Purdue University, West Lafayette, Indiana, United States of America; 8 Department of Statistics, Purdue University, West Lafayette, Indiana, United States of America; 9 Department of Statistics, Carnegie Mellon University, Pittsburgh, Pennsylvania, United States of America; 10 School of Informatics, Indiana University, Indianapolis, Indiana, United States of America; 11 Medicinal Chemistry and Molecular Pharmacology, Purdue University, West Lafayette, Indiana, United States of America; 12 Department of Biochemistry, University of Nebraska, Lincoln, Nebraska, United States of America; Alexander Flemming Biomedical Sciences Research Center, Greece

## Abstract

**Background:**

Methamphetamine (METH), an abused illicit drug, disrupts many cellular processes, including energy metabolism, spermatogenesis, and maintenance of oxidative status. However, many components of the molecular underpinnings of METH toxicity have yet to be established. Network analyses of integrated proteomic, transcriptomic and metabolomic data are particularly well suited for identifying cellular responses to toxins, such as METH, which might otherwise be obscured by the numerous and dynamic changes that are induced.

**Methodology/Results:**

We used network analyses of proteomic and transcriptomic data to evaluate pathways in *Drosophila melanogaster* that are affected by acute METH toxicity. METH exposure caused changes in the expression of genes involved with energy metabolism, suggesting a Warburg-like effect (aerobic glycolysis), which is normally associated with cancerous cells. Therefore, we tested the hypothesis that carbohydrate metabolism plays an important role in METH toxicity. In agreement with our hypothesis, we observed that increased dietary sugars partially alleviated the toxic effects of METH. Our systems analysis also showed that METH impacted genes and proteins known to be associated with muscular homeostasis/contraction, maintenance of oxidative status, oxidative phosphorylation, spermatogenesis, iron and calcium homeostasis. Our results also provide numerous candidate genes for the METH-induced dysfunction of spermatogenesis, which have not been previously characterized at the molecular level.

**Conclusion:**

Our results support our overall hypothesis that METH causes a toxic syndrome that is characterized by the altered carbohydrate metabolism, dysregulation of calcium and iron homeostasis, increased oxidative stress, and disruption of mitochondrial functions.

## Introduction

The term “systems biology” refers to the interdisciplinary study of complex interactions that give rise to the function and performance of a particular biological system. Currently, transcriptomics, proteomics, and metabolomics are the principal technology platforms that provide useful data for systems biology analyses. Data from these various platforms are integrated to reveal how cellular systems respond to xenobiotics like plant defense compounds, food ingredients [1], [2], pesticides, and drugs, thereby providing insights into how animals are affected by xenobiotic challenges and possible ways to alleviate their negative biological effects.

When used in combination with model organisms, xenobiotic challenges also provide an opportunity to test analytical approaches based on systems biology. For example, METH is a central nervous system stimulant that is increasingly abused, especially by teenagers and young adults, and that causes acute and chronic side effects in multiple organ systems [1], [2]. However, most molecular studies on the impact of METH have focused on brain tissues [3], [4], [5], including recent work by Chin *et al* [6] using combined proteomic and transcriptomic analyses. However, to our knowledge, there are no systems biology analyses of the impact of METH on whole organisms. In terms of a model organism, *Drosophila melanogaster* has one of the best-defined genomes among insects [7] and a robust set of available mutants, making it an excellent system with which to elucidate the mechanisms underlying the genomic, proteomic, and metabolomic whole-organism responses to xenobiotics and to obtain follow-up validation through mutant analysis. Moreover, METH influences evolutionarily conserved pathways shared by *Drosophila* and mammals (*e.g.*, oxidative phosphorylation). Importantly, xenobiotic perturbations of conserved molecular pathways have the potential to generate similar cellular- and organism-level responses across species.

Here we report that the administration of METH to *Drosophila* causes a METH-induced cytotoxic syndrome. Consumption of this drug has been associated with several disorders in humans and in animal models, including defects in the male reproductive system, changes in blood sugar levels, induction of oxidative stress, neurological damage, heart disease, reduction of mitochondrial energy production, increased lactic acid build up, and apoptosis in multiple tissues [8], [9], [10], [11], [12], [13], [14], [15]. METH syndrome produces changes in cellular energy metabolism that appear to be consistent with a Warburg effect, which is characterized by high levels of glycolysis (followed by lactic acid fermentation) and decreased oxidative phosphorylation in the mitochondria, even under aerobic conditions [16], [17]. These metabolic changes, however, could also be consistent with hypoxia. The Warburg effect has not previously been associated with METH syndrome.

Using a systems biology approach, we present a mechanism-based model to describe the molecular impacts of METH on cellular pathways, followed by a mutant analysis of key METH-responsive genes including those with known and previously unknown function. We also determined that dietary trehalose reduced METH toxicity in *Drosophila*. Trehalose is an antioxidant and the major blood sugar in insects [18], [19]. Combined results from systems biology and mutant analyses have the potential to give us an in-depth understanding of the impact of xenobiotics on cellular and organismal systems.

## Results and Discussion

### Systems biology elucidates complementary aspects of the METH syndrome

#### Gene pathways detected by microarray

To elucidate potential pathways responsive to METH, we analyzed microarray data, comparing control and METH-treated *Drosophila* males through Gene Ontology (GO) system categorizations (http://www.geneontology.org) and the Kyoto Encyclopedia of Genes and Genomes (KEGG) pathway analyses (http://www.genome.ad.jp/kegg/). Genes with a *p* value smaller than 0.008 and an absolute fold change greater than 1.5 were considered significant and used for the analyses. The top eight pathways were (i–v) detoxification/drug metabolism pathways, (vi) glutathione metabolism, (vii) glycolysis/gluconeogenesis, and (viii) purine metabolism (Table S1). In total, we observed 229 differentially transcribed genes and 34 potential pathways, some of which are consistent with METH syndrome (*e.g.*, energy-associated pathways) and known specific responses to METH (such as tyrosine metabolism); METH is known to lead to long-term decreases in the activity of dopamine transporter and tyrosine hydroxylase [20].

#### Proteomic analysis

Initially, we identified 226 spectral peaks that were differentially expressed after METH treatment (*p*<0.05 and a fold-change of >2). We were able to identify the associated peptides for 87 of the original 226 peaks (SpectrumMill peptide score of >6 and SPI% >60%) (Table S2). Because multiple peptides were observed for a single protein, only 72 proteins were identified: 33 increased in abundance, 35 decreased, and 4 proteins (CG4169-PA; ATP synthase- CG11154-PA, isoform A; enolase CG17654-PE, isoform E; and, MyHC) (Table S2) had associated peptides that were for unknown reasons both increased and decreased (they were probably associated with different isoforms of the same protein). The 72 differentially expressed proteins were then categorized according to their involvement in 26 pathways, including those relating to heart and skeletal muscles, oxidative stress, energy, oxidative phosphorylation, and spermatogenesis. All 26 pathways are known to be associated with METH responses in mammals (Table S2).

#### Impact of METH on combined transcriptome and proteome pathways

**Figure 1:**
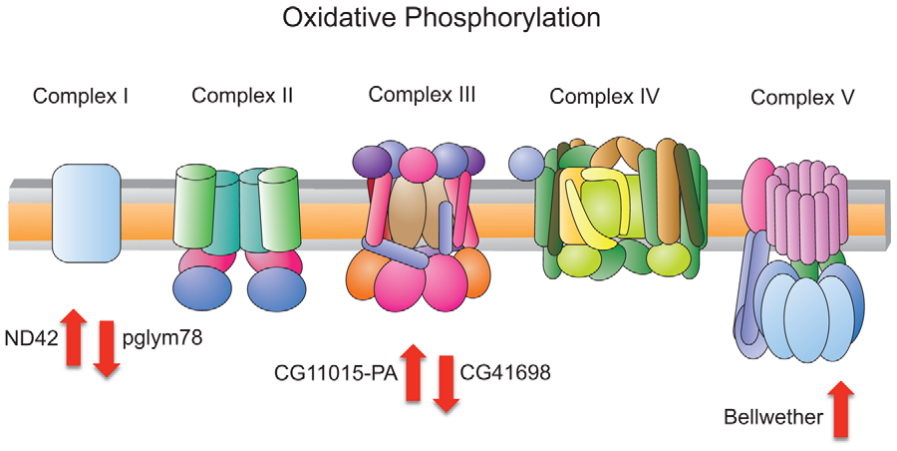
Changes in abundance of key proteins associated with oxidative phosphorylation. METH exposure results in changes in the abundance of key proteins in the mitochondrial electron transport chain (ETC), including complexes I, III and V. The direction of the red arrow indicates specific proteins that either increased (up) or decreased (down) in abundance as a result of METH exposure. Protein expression data is also given in Table S2. The figure for ETC was adapted from http://www.genome.jp/kegg-bin/highlight_pathway?scale=1.0&map=map00190&keyword=oxidative.

The database for annotation, visualization, and integrated discovery (DAVID) analysis indicated that a large number of differentially expressed proteins were involved in glycolysis and oxidative phosphorylation (Figure 1; Figure S1 and Table S3). Because only one common protein/gene, glycerol-3-phosphate dehydrogenase (GPDH), was present in both the proteomic and gene expression data in the METH-treated flies, we performed multiple pathway analyses in which these two “omic” data sets were combined. Although each of these analyses revealed somewhat different pathways, all the pathways identified were consistent with METH syndrome (Figures S2 & S3). A process network analysis of the proteomic and microarray data revealed that of the top 10 networks impacted by METH, 8 were associated with skeletal muscle, cardiac muscle, cytoskeleton systems, and oxidative stress (Figure S2). Statistically significant test results for genes or proteins enriched in pathways performed with DAVID software were obtained for several pathways related to changes in both the microarray and proteomics experiments (Figure S3). These pathways include glycolysis, oxidative phosphorylation, hormonal pathways and cytoskeletal remodeling.

### Biological relevance of pathways associated with METH syndrome

The pathways that we observed in our transcriptomic and proteomic analyses are consistent with the known effects of METH on biological systems, including proteases, detoxification enzymes, oxidative stress and iron homeostasis (See Text S1). However, because we analyzed whole-organisms, as compared to previous studies on brain tissue [3], [4], [5], we observed proteins that had not been previously associated with METH-induced responses, including certain proteins involved in the electron transport chain, muscle formation/homeostasis, and spermatogenesis.

### Mitochondrial electron transport chain (ETC)

METH has been previously shown to affect the mitochondrial electron transport chain (ETC) [21]. We observed changes in the abundance of proteins associated with the ETC, corroborating the work of others who have observed that METH inhibits the ETC in mice and rats [9], [22]; Burrows and co-workers observed the inhibition of cytochrome oxidase activity [9] (complex IV of the ETC) in rat brains after METH treatment, and Brown *et al.* [23] observed that succinate dehydrogenase (complex II of the ETC) was inhibited by METH in the striatum of rats. We observed changes in the prevalence of multiple proteins in the oxidative phosphorylation pathway beyond complex II and IV (Figure S3), including those associated with complex I (phosphoglycerate mutase [*pglym78*] and NADH dehydrogenase [ubiquinone] 1 alpha subcomplex subunit 10 [*ND42*)]), complex III (CG3815 with ubiquinol-cytochrome-c reductase activity), and complex V (ATP synthase subunit alpha [also known as *bellwether* in *Drosophila*]) of the ETC (Figure 1). Alaux *et al.* observed that aggression in bees is associated with reduced enzyme activity for complexes I (NADH dehydrogenase), IV (cytochrome c oxidase), and V (ATP synthase) [24] (Table S2). Whether METH-induced aggressive behavior is associated with the oxidative phosphorylation pathway has not been determined.

#### Proteins related to muscle and heart disease

METH causes muscle loss [25], [26] and heart failure in humans [27], however, little is known about the molecular mechanisms by which METH causes this effect. In *Drosophila*, we observed that the concentrations of numerous muscle-associated proteins changed in response to METH exposure. For example, dynein heavy chain and troponin c decreased 6- and 2-fold, respectively, and tropomyosin protein levels increased 10-fold (Table S2). Additionally, we observed that three MyHC peptides increased approximately 10-fold (Table S2) and two other MyHC peptides decreased 8-fold with METH treatment, suggesting that METH likely disrupts normal muscle physiology in *Drosophila*. This is supported by the observation that changes in the relative mRNA expression of alpha - and beta-myosin heavy chain (MyHC) are associated with chronic heart failure in humans [28].

#### Spermatogenesis- and ejaculation-related proteins

METH causes sexual dysfunction in mammals, inhibiting sperm motility [29], and amphetamines, which are structurally similar to METH, retard ejaculation in humans [30]. Although METH is known to have negative effects on male fertility [29] little is known regarding the molecular impacts of METH on spermatogenesis. In our study, we detected seven METH-responsive genes and proteins that are associated with reproductive functions in males (Tables S2 & S3), some of which are evolutionarily conserved in humans.

The transcript of *CG11893*, which was up-regulated in METH-treated flies, is associated with the *Androcam* gene; this gene encodes a protein abundantly expressed in the cones of the testes in *Drosophila* [31]. The C-domain of *Androcam* binds calcium and has 67% homology with a mammalian calmodulin [32] protein that has testes-specific calcium signaling functions [31]. *CG11893*, which is associated with *poe*, has protein-binding functions (UniProtKB).

The protein CG32542, which is over-expressed in METH-treated flies, interacts with *ocn* (iHOP- http://www.ihop-net.org/) [33], a testes-specific gene [34]. The protein CG3815 was under-expressed in METH-treated flies and interacts with *sneaky*, a testes-expressed gene involved in sperm exocytosis in *Drosophila* [35]. Fertilization typically involves membrane fusion between sperm and eggs. In *Drosophila*, however, sperm enter eggs with membranes intact, and the membranes are broken down in the egg; only then are the sperm activated. Mutations in *sneaky* can impair this process, resulting in male sterility due to impaired sperm plasma membrane breakdown [35]. *Sneaky*-like genes have also been detected in humans [35].

Tubulins also have an important function in spermatogenesis [36]. We observed that the alpha-tubulin 84B protein, a component of the spermatozoa cytoskeleton involved in spermatid development [37], decreased in METH-treated flies. Two mitochondrial proteins—porin, which decreased, and *bellwether*, which increased—were identified in flies exposed to METH. Porin is localized in the outer mitochondrial membrane of germ cells in the testes and in the spermatozoa in *Drosophila* [38]. The mitochondrial ATP synthase subunit alpha of complex V (*bellwether*) is also involved in spermatogenesis and is associated with male sterility [39]. In addition, the expression of the predominant ejaculatory bulb protein (PEB-me), a component of the mating plug in *Drosophila* [40], [41], increased 10-fold in response to METH treatment (Table S2). Moreover, the germ cell nuclear-like factor (GCNF) was identified in METH-treated flies; this potential transcription factor binding motif (TFBM) is associated with germ cells (Figure S4, S5, S6, Text S1, Table S4 and Table S5). GCNF targets several genes involved in sperm maturation.

### Metabolomic profiling and dietary trehalose

Previous studies in mammals have shown that METH toxicity is interrelated with metabolism in the brain and sugar levels in the blood [3], [4], [5], [42], [43]. Thus, we also investigated changes in whole organism sugar levels in *Drosophila* in response to METH. Using gas chromatography/mass spectrometry (GC/MS), we observed decreased trehalose levels in METH-exposed *Drosophila* (*p*<0.0001; Figure S7). That trehalose acts as an antioxidant [18], [44], and thus is itself oxidized, could account for reduced trehalose levels. Because trehalose is the major blood sugar in insects, decreased levels of trehalose could also reflect either higher metabolic rates resulting from a METH-induced increase in physical activity or increased carbohydrate consumption resulting from increased glycolysis.

We found that METH-treated flies fed a diet containing trehalose or sucrose lived longer than flies treated only with METH (*p*<0.01 and *p*<0.05, respectively; Table 1). In contrast, sorbitol, a sugar alcohol that is not well metabolized by *Drosophila* [45], had no impact on METH toxicity. These results suggest that METH-toxicity is interrelated with carbohydrate metabolism, corroborating previous findings where it has also been observed that supplementation with cofactors of energy metabolism attenuates the toxicity of METH [8], [46]. Interestingly, human METH addicts often imbibe large amounts of sugary soft drinks [47]; such dietary studies in *Drosophila* lead us to question whether sugar intake in humans helps to alleviate the toxic effects of METH.

**Table 1:** The lethal time 50 (LT_50_) and 95% confidence interval (C.I.) of *Drosophila melanogaster* fed on methamphetamine (METH), and different sugars (including trehalose, sucrose, and sorbitol) plus METH.

Treatment	LT_50_ (h)	95% C. I. (h)	*P* value*
METH 0.6%	50.40	45.19–55.58	–
Trehalose 0.189M+METH 0.6%	91.99	80.31–112.04	<0.01
Sucrose 0.189M+METH 0.6%	71.88	67.34–77.03	<0.05
Sorbitol 0.189M+METH 0.6%	62.68	56.74–69.01	N.S.

*Comparisons were between the LT_50_s of the given sugar plus METH treatment versus the METH only treatment. N.S. stands for not significant. The LT_50_s and treatment comparisons were performed using SAS (Cary, NC).

### Oxidative stress

We observed multiple genes and proteins associated with an oxidative stress in METH-treated *Drosophila*; METH also induces oxidative stress in mammals [6]. Oxidative stress has been linked to many pathways, including alcohol dehydrogenase (ADH) activity [48], actin reorganization [49], and the inhibition of hexokinase activity in rabbit erythrocytes [50]. Aconitase also helps to regulate resistance to oxidative stress and cell death in two plant species, *Arabidopsis thaliana* and *Nicotiana benthamiana* [51]. Consistent with the hypothesis that the METH-treated flies are experiencing oxidative stress, we observed decreases in alcohol dehydrogenase (ADH) and aconitase, as well as increases in hexokinase and actin (Table S2).

Oxidative stress also causes thiol oxidation in the glyceraldehyde-3-phosphate dehydrogenase (GAPDH) of *Staphylococcus aureus* [52] and increases GAPDH transcript levels in rabbit aortas [53]. Perhaps because of the oxidative stress involved in exposure to METH, we observed a 10-fold increase in GAPDH in the treated flies (Table S2). GAPDH belongs to an evolutionarily conserved protein family, the aldehyde dehydrogenases; these play a key role in stress responses, including oxidative stress [54], [55].

**Figure 2:**
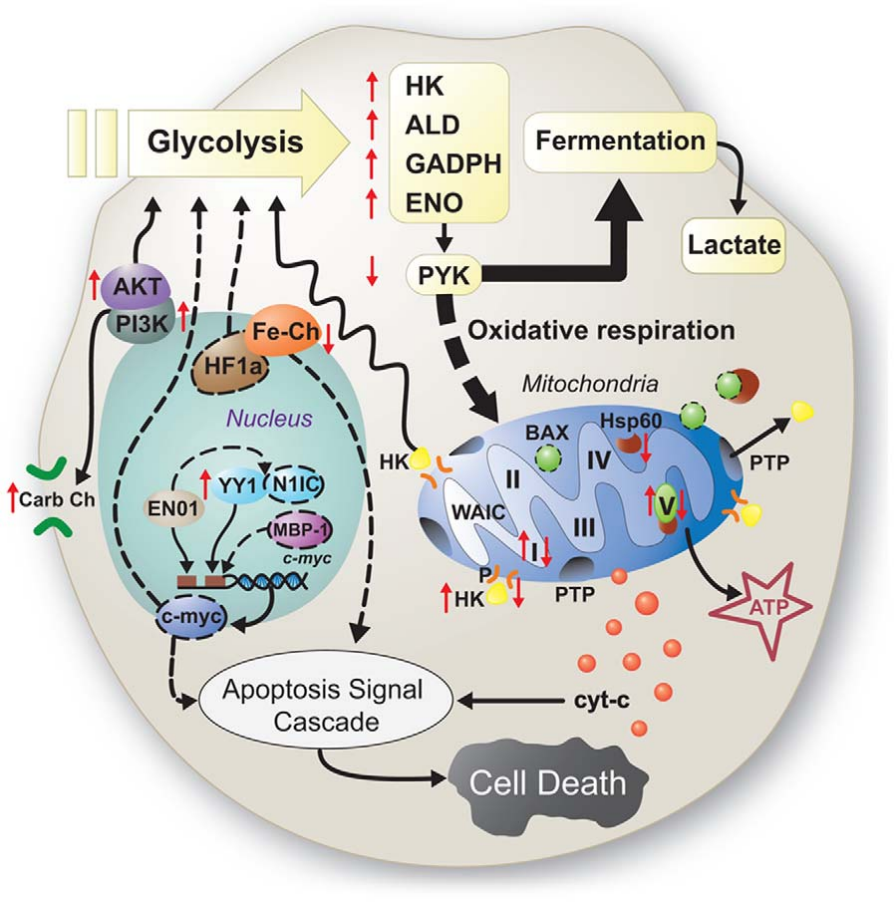
Changes in energy metabolism and apoptosis in response to METH treatment. In METH metabolic syndrome, key glycolytic enzymes [*i.e.*, HK (hexokinase), ALD (aldolase), GAPDH (glyceraldehydes 3-phosphate dehydrogenase), and ENO (enolase)] are up-regulated (upward-pointing red arrows), presumably leading to higher glycolytic flux. PYK (pyruvate kinase), however, was down-regulated (downward-pointing red arrow), diverting pyruvate away from oxidative respiration towards fermentation to lactic acid. Mitochondrial degradation (see Figure 3 for detailed discussion) and dysregulation of the electron transport chain (see Figure 1) may also contribute to decreased oxidative respiration. Hsp60 binds to BAX, preventing apoptosis. Thus, the down-regulation of Hsp60 observed with METH exposure may potentially increase free BAX, leading to apoptosis. Multiple genes known to transcriptionally regulate glycolysis and/or apoptosis were differentially regulated during METH exposure, including (i) AKT and PI3K, which were up-regulated, enhancing glycolysis and carbohydrate transport across the cellular membrane, and (ii) iron chelators (Fe-Ch), which were down-regulated and are known to interact with HF1a (genes not detected experimentally, indicated by dashed lines). In addition, ENO1 and YY1, which suppress and activate, respectively, the transcription of c-Myc, were both up-regulated, suggesting a loss of fine control over c-Myc. c-Myc in turn regulates the transcription of many other genes including those involved in apoptosis and glycolysis.

Our data also suggest that METH induces multiple pathways associated with the generation of reactive oxygen species (ROS) (Figure 2). Flies challenged with METH differentially expressed multiple genes and exhibited changed protein levels associated with the mitochondrial ETC, potentially leading to ROS formation. High levels of P450s, which we observed in METH-treated *Drosophila*, in some cases can also lead to the generation of ROS during detoxification and catabolism, which can cause downstream ROS formation. For example, the degradation of the endoplasmic reticulum [56], the main Ca^2+^ storage areas of the cell, disrupts Ca^2+^ homeostasis [57]. Dysregulation of Ca^2+^ homeostasis can lead to cell death, especially under conditions of oxidative stress, because high levels of cytoplasmic Ca^2+^ can cause the formation of nonspecific pores, known as permeability transition pores, in the inner mitochondrial membrane. These pores may cause the mitochondria to swell massively, depolarize, and generate ROS, leading to cell death [58]. Additionally, the tumor suppressor p53 protein is induced by ROS, leading to apoptosis following treatment with METH [59], [60], [61].

ROS react with and cause damage to cellular macromolecules, including DNA and membrane phospholipids. For example, ROS can chemically modify and fragment DNA [62], potentially leading to genetic mutations. Helicases play a central role in repairing DNA damage caused by UV-light and ROS. These proteins also reverse the DNA damage associated with replication errors and thus help maintain genomic stability. Our observation that one helicase was down-regulated (Table S2) is consistent with the hypothesis that METH causes oxidative DNA damage [63].

For membrane phospholipids, ROS cause lipid peroxidation, a process that may result in the degradation of cellular and mitochondrial membranes. The resulting change in mitochondrial membrane permeability triggers a signaling cascade that causes the release of cytochrome c into the cytoplasm, triggering the downstream caspase-dependent apoptosis [64]. Mitochondrial degradation might contribute to higher levels of free Ca^2+^ that can in turn activate phospholipase A, promoting the hydrolysis of membrane phospholipids, which further disrupts the cellular compartmentalization of Ca^2+^. Furthermore, P450 enzyme systems, which were up-regulated in METH-exposed *Drosophila*, in some cases modulate Ca^2+^ channels that in turn trigger fluxes of Ca^2+^ [65]; these further increase levels of intracellular free Ca^2+^. An imbalance in Ca^2+^ homeostasis due to oxidative stress is also an important factor in heart disease [66].

Iron also plays a role in responses to oxidative stress. Free iron, through the Fenton reaction, can produce harmful free radicals from hydrogen peroxide [67]. Ferritin, a major regulator of iron homeostasis [68], chelates iron and prevents the Fenton reaction. Therefore, it is reasonable to hypothesize that the down-regulation of ferritin that we observed with METH-exposure could enhance the generation of ROS.

### Integrating the effects of METH on cellular pathways

**Figure 3:**
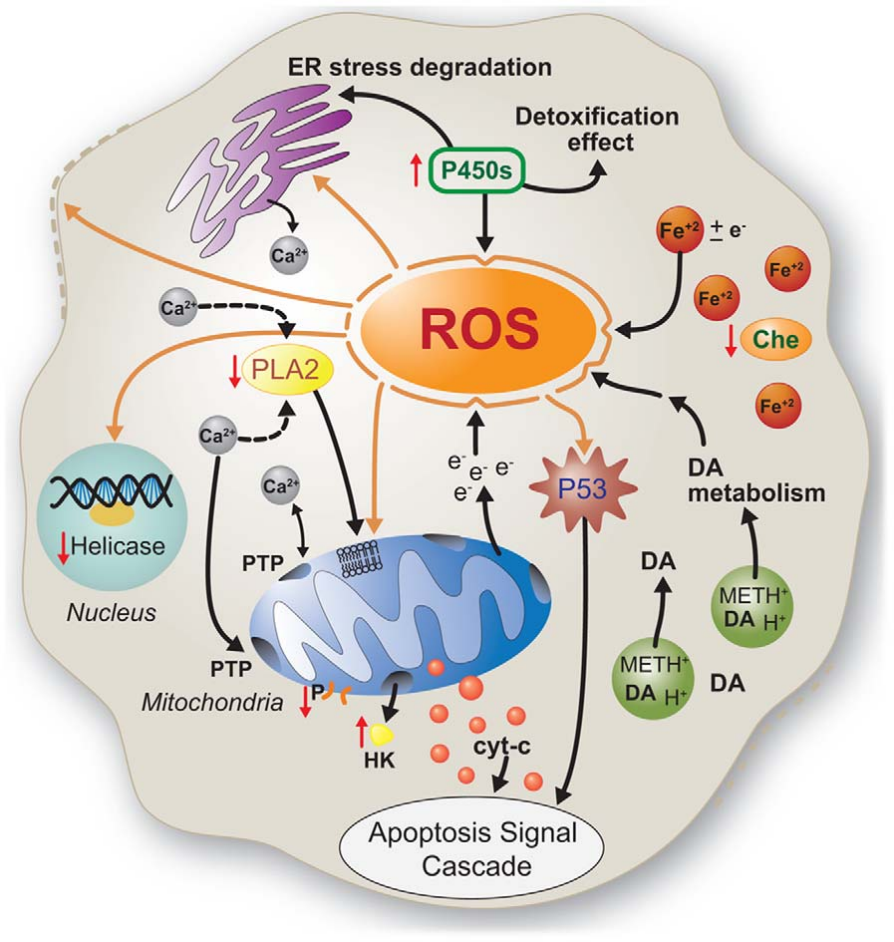
METH exposure may promote oxidative stress through multiple mechanisms. First, cytochrome P450s are up-regulated (upward-pointing red arrow) to potentially detoxify METH but in some cases also produce reactive oxygen species (ROS) byproducts. Second, METH, a weak base, is known to alkalinize dopamine (DA)-containing vesicles, promoting DA release into the cytosol. Cytosolic DA is rapidly degraded, resulting in ROS byproducts. Third, iron chelators are down-regulated (downward-pointed red arrow), potentially increasing the concentration of free iron, a known source of ROS. Fourth, degradation of the mitochondria, potentially resulting from (i) the ability of high cytosolic Ca^2+^ to promote the formation of permeability transition pores (PTP), (ii) increased membrane hydrolysis by Ca^2+^-dependent phospholipase A_2_ (PLA2) in the presence of increased Ca^2+^, (iii) the direct effects of ROS on mitochondrial integrity, and (iv) the potential effects of altered HK/porin ratio - HK detachment from mitochondria on PTP formation. This may cause the mitochondria to uncouple and result in ROS production. The cellular targets of oxidative stress, indicated by orange arrows, include membrane phospholipids; p53, a gene that regulates apoptosis; and DNA.

METH-treated *Drosophila* differentially expressed multiple genes, proteins, and pathways associated with both hypoxia and the Warburg effect (aerobic glycolysis) [16], [17] (Figure 3). In the mammalian liver, pyruvate kinase is positioned at a key branch-point in glucose metabolism, and a high expression level of this protein is correlated with the aerobic status of the cell [69]. This enzyme was down-regulated in the METH-treated flies, suggesting that METH either induces an anaerobic response or a Warburg-like effect [70] or some third hitherto unknown process.

The heat shock protein 60 (Hsp60), primarily a mitochondrial Hsp, is also an indicator of the aerobic status of the cell and is involved in the apoptotic response [70], [71]. Hsp60 is expressed at high levels in normal cells, but hypoxia decreases its expression and/or changes its cellular distribution [70], causing apoptosis. Indeed, decreasing the level of Hsp60 in cardiac myocytes was sufficient to cause apoptosis [71]. Hsp60 forms a macromolecular complex with the pro-apoptotic protein BAX, which blocks the ability of BAX to translocate to the mitochondria and to promote apoptosis *in vivo* [70]. The reduced expression of the Hsp60 protein in METH-treated flies, which we observed, supports the idea that METH induces hypoxia.

Levels of the mitochondrial ATP synthase, *bellwether*, increased with METH exposure. The over-expression of the subunits of the catalytic core of the mitochondrial F_(0)_–F_(1)_ ATP synthase complex, including the alpha-subunit, are correlated with apoptosis [72]. Up-regulation of these proteins apparently causes a transient increase in intracellular ATP levels, which is necessary for apoptosis [72]; pharmacologically inhibiting ATP synthase blocks apoptosis. Apoptosis is induced in response to a specific signal that indicates an imbalance between aerobic and anaerobic ATP biosynthesis [69].

Several oncogenes have been implicated in the Warburg effect, including the serine-threonine kinases (AKT) that enhance glucose uptake and aerobic glycolysis in cancer cells [73] and are able to do so independently of hypoxia-inducible factor (HIF-1); the levels of two AKT proteins significantly changed in the METH-treated flies. AKT mobilizes glucose transporters to the cell surface to enhance glucose uptake and activates hexokinase (HK) [73], a protein that was over-expressed in the METH-treated flies. Elstrom and co-workers reported that through these effects, AKT is able to enhance glycolytic flux without affecting mitochondrial oxidative phosphorylation, thereby presumably contributing to the Warburg effect [73]. Moreover, the AKT and phosphatidylinositol triphosphate kinase (PI3K) protein levels were up-regulated in METH-treated flies. The PI3K-AKT signaling pathway promotes cell growth, increases glucose uptake, influences cell cycle progression, and prevents apoptosis through multiple mechanisms [73].

The transcription factor c-Myc, a known oncogene, regulates the cell cycle, differentiation, apoptosis, metabolism, and cellular responses to oxidative stress. Typically, the expression of c-Myc is tightly regulated by multiple transcriptional activators and repressors. In METH-treated *Drosophila*, however, multiple genes that regulate c-Myc were differentially expressed. For instance, YY1 transcription factor, which has previously been associated molecular responses to oxidative stress and heart disease [74], [75], activates the transcription of Notch 1 transcription factor (N1IC) [76]. Subsequently, the N1IC-YY1 complex binds to the major promoter of the c-Myc gene and activates its expression [76]. In addition, enolase, which was up-regulated in METH-treated flies, and promoter binding protein 1 (MBP-1), which results from an alternative translation initiation codon of the enolase gene, are transcriptional repressors of c-Myc [77]. The simultaneous up-regulation of transcriptional activators and repressors suggests that METH disrupts the fine control of c-Myc.

Interestingly, c-Myc has been associated with the direct activation of aerobic glycolysis in human cancers. Numerous METH-responsive glycolytic genes and proteins detected in our microarray and proteomic analysis are known to interact with c-Myc (Figure S3). For example, c-Myc activates many glycolytic genes, including hexokinase (HK) and enolase [78], [79], both of which were over-expressed in METH-treated flies.

Increased glycolytic activity requires increased glucose uptake via glucose transporter proteins and the increased expression of glycolytic enzymes. METH treatment induced changes in the flies' expression of glucose transporters, adolase (Ald), and glyceraldehyde-3-phosphate dehydrogenase (GAPDH) (Table S2). Ald and GAPDH are associated with the production or elimination of glyceraldehyde-3-phosphate in the process of glycolysis or gluconeogenesis, and are differentially expressed under many physiological conditions, such as cancer, hypoxia, and apoptosis [80], [81].

The Warburg effect is also associated with other apoptotic pathways, including one that is induced by voltage-dependent anion channels called porins [82]. Porins are located in the outer mitochondrial membrane and have been widely implicated in the initiation of the mitochondria-mediated intrinsic pathway of apoptosis. Furthermore, porins have been characterized as an important component in the distribution of mitochondrial membrane cholesterol, which in turn is associated with aerobic glycolysis [82]. Importantly, porin is a binding partner for HK, a protein associated with the Warburg effect [82], [83]. The increased affinity of porin to HK increases cellular access to ATP, which increases use of the glycolytic pathway. Therefore, the direct binding of HK to porins and the involvement of porins in cell death suggest that interactions between HK and porin are a component of apoptosis regulation by HK [83]. In METH treated flies, porins were under-expressed and HK protein increased more than 10-fold. It is possible that alterations in the HK-porin relationship influence the apoptotic pathway. This prediction is supported by a recent report that the over-expression of HK in human cells suppressed cytochrome c release and apoptotic cell death [83]. In addition, a single mutation in porin decreased HK binding, diminishing the protection that HK offers against cell death. Alternatively, Chiara and co-authors suggested that HK detachment (independent of porins) from mitochondria induces the PTPs that cause mitochondrial degradation and apoptosis [84]; furthermore, Shoshan-Barmatz and co-authors observed that over-expression of HK corresponds to an anti-apoptotic defense mechanism used by malignant cells [85].

Both enolase (which synthesizes phosphoenol pyruvate) and calcium ion homeostasis are also involved in apoptosis. Some cancers, such as neuroblastoma, have an associated genomic deletion that corresponds to the enolase gene *(ENO1)*. When a functional copy of enolase is transfected to this type of cancer cell, it causes apoptosis [86]. Additionally, METH-treated flies up-regulated enolase 10-fold. Calcium also has an important role in signaling pathways associated with cell death and drug resistance [87]. The cytosolic Ca^2+^ concentration is controlled by interactions among transporters, pumps, ion channels, and binding proteins. Consistent with these observations, METH treatment affected the expression of several calcium-binding proteins (Table S2). *Drosophila* possessing a mutant Giiispla2 gene (Table S6), which encodes a Ca^2+^ binding protein, was more susceptible than the *w^1118^* control to METH, suggesting that the disruption of Ca^2+^ homeostasis affects apoptosis. Alternatively, increased susceptibility of the Giiispla2 mutants might be related to altered arachidonic acid metabolism (Table S1).

Iron chelators also activate a hypoxia stress-response pathway. We found that the METH syndrome decreases the expression of ferritin and aconitase (Table S2). Iron chelators induce the expression of hypoxia-inducible factor-1 (HIF-1) and glycolytic enzymes [16], [88]. These studies highlight the diversity of cellular responses to iron chelators and suggest that these multifunctional antiapoptotic agents may enhance survival by suppressing ROS generation as well as by inducing glycolytic enzymes, such as aldolase and enolase, and glucose channels. Changes in the expression of these genes are observed in the METH syndrome (Table S2 & Table 1).

In summary, our observations indicate that METH impacts pathways associated with hypoxia and/or the Warburg effect, pathways in which cellular energy is predominantly produced by glycolysis rather than by oxidative respiration. These results are consistent with the fact that METH use is associated with the formation of lactic acid (Figure 2) [8]; lactate dehydrogenase mRNA was over-transcribed 1.8-fold in METH-treated flies (*p*<0.01). Further work is required to validate the role of these pathways in response to METH. However, an approach based on systems biology, validated by mutant analysis or feeding studies or both, has the potential to accelerate the discovery of the molecular effects of drugs and potential dietary factors that can alleviate the effects of drugs. Additionally, two mutants for two separate genes with previously unknown function (CG14280 and CG7796) were more susceptible to METH (as compared to the non-mutant control flies), raising the possibility that systems biology in combination with targeted mutant analysis could be useful for elucidating other unknown aspects of METH toxicity (Table S6).

## Materials and Methods

### *Drosophila melanogaster* stock

The *w^1118^* strain was obtained from Dr. Misha Ludwig (University of Chicago) and reared on the Formula 24® *Drosophila* diet (Carolina Biological Supply, Burlington, NC) at 22–23°C and 60–70% humidity.

### METH bioassays

For microarrays and proteomic and metabolomic experiments, virgin male flies were collected during the sixth to seventh hours following eclosion from the pupae [89] and cultured for 5 days. Twenty of these flies were then placed on a standard fly diet (control) or a diet supplemented with 0.6% (w/v) METH (Sigma, M8750, St. Louis, MO) for 24 h. Three biological replicates were performed for each experiment (for a total of six samples). At the end of the 24 h feeding period, the 20 flies were collected, flash-frozen in liquid nitrogen, and stored at −80°C. These samples were subsequently used in the DNA oligoarray experiments, proteomic or metabolite analyses.

For toxicology experiments of sugar feeding treatments, virgin male flies were collected as aforementioned, and cultured for 5 days. Nine of these flies were placed on one of following diets: 1) 0.6% (w/v) METH (methamphetamines), 2) 0.6% METH+5% (0.189M) trehalose (Sigma, T9449, St. Louis, MO), 3) 0.6% METH+5% (0.189M) sucrose (Sigma, 84097, St. Louis, MO), and 4) 0.6% METH+0.189M sorbitol (Sigma, W302902, St. Louis, MO). Three biological replicates were performed for each treatment.

For toxicology experiments of mutant flies, 3–5-day-old male flies were collected to determine the lethal time 50 (LT_50_). Five of these flies were placed on either a standard fly diet (control) or a diet supplemented with 0.6% (w/v) METH. Six biological replicates were performed for each mutant. Mutant flies were ordered from Bloomington *Drosophila* Stock Center at Indiana University.

### Microarray experiment

Total RNA was extracted using Trizol reagent for each replicate (Invitrogen Life Technologies, Carlsbad, CA). Three replicates each were used for control and METH-treated flies. DNA contamination was removed by DNaseI set (Qiagen Inc., Valencia, CA) followed by another step of Trizol extraction to remove DNase. Affymetrix oligoarray experiments were performed as described in Pedra *et al.* [90]. Amplified cRNA was hybridized to Affymetrix *Drosophila* Genome 2.0 Arrays (Affymetrix, Santa Clara, CA); this array allows for the potential analysis of over 18,500 different *Drosophila* transcripts. Gene-chip-operating software (GCOS 1.4) was used to quantify the images. Microarray data were deposited in the Gene Expression Omnibus (GEO) database with accession number GSE16198.

### Quantitative real time PCR (qRT-PCR)

cDNA was synthesized using 1 µg of total RNA with iScript cDNA kit (Bio-Rad, Hercules, CA) in a 20 µl reaction. Primers (Table S7) were designed by primer3 online and Genscript Real-Time PCR Primer Design (http://fokker.wi.mit.edu/primer3/input.htm & https://www.genscript.com/ssl-bin/app/primer). The 2× iQ SYBR Green Supermix was purchased from Bio-Rad (Hercules, CA). qRT-PCRs were performed on an iCycler Thermal Cycler with an annealing temperature of 60°C and 30 cycles. Each cDNA sample has triplicates. The statistical analyses of the relative gene expression level were performed using the SAS TTEST (SAS Institute Inc., Cary, NC). AFFX-Dros-ACTIN_M_r was used as the reference gene, and the significance analysis of the microarray (SAM) and transcriptional analyses of 21 genes were performed on it.

### Metabolomic and GC/MS procedure

For metabolite extraction, each sample was removed from the freezer and 200 µL of 100% ethanol was added to each tube. A sterile plastic pellet pestle was used to grind each sample for 3 minutes. The samples were then placed onto a heating block set to 80°C. After 10 minutes, 400 µL of methanol/water (50∶50 v/v) mixture was added and vortexed for 30 minutes at room temperature. Once the extraction was complete, the tubes were centrifuged at 13,000 g for 10 minutes. The supernatant was transferred to a new tube and dried using a rotary evaporation device at 43°C for 3 hours. The samples were derivatized with 20 µL of O-Methylhydroxylamine-HCl solution (20 mg/mL anhydrous pyridine) by heating them to 60°C for 30 minutes. Subsequently, 30 µL of MSTFA labeling reagent was added to each tube and incubated at 60°C for one hour. After the heating process, each sample was allowed to cool to room temperature and then transferred to a glass autosampler vial.

The GC-MS instruments used were the Pegasus 4D GCxGC-TOFMS from Leco Corp (St. Joseph, MI), an Agilent 6890N GC, and an Agilent 7683B Series autosampler. The first dimension column was a HP-5MS phase, 30 m length, 0.250 mm I.D., 0.25 um film. The second dimension column was a DB-17 phase, 1 m length, 0.100 mm I.D., 0.10 um film. Both columns were from Agilent. A 3 µL injection was made for each sample using helium as a carrier gas at a flow rate of 1 mL/minute.

The front inlet split was set to 20 and the inlet temperature was 280°C. The temperature gradient was as follows: 50°C for 0.20 minutes, ramped 10°C/min to 250°C and held for 10 minutes, ramped 25°C/min to 300°C and held for 5 minutes. The second dimension temperature profile was exactly the same, only +20°C. The transfer line between GC and MS was set to 250°C. The MS had a solvent delay of 150 seconds. Data were collected from 30–1000 m/z with an acquisition rate of 100 spectra/second. The detector voltage was 1700 and electron energy was −70 V. The ion source was set to 200°C. All data were processed using Leco ChromaTOF software (Version 3.32). Area and height calculations were based on the 73 ion. Standard curves for the trehalose metabolite were generated, using an equimolar mixture of standards at 5 concentrations (0.5, 0.25, 0.05, 0.025, and 0.005 µmols).

### Statistical analyses for FDR in microarrays, cellular metabolomics, and toxicity

The oligoarray data were transformed by log base 2 and normalized by AFFX-Dros-ACTIN_M_r, and then analyzed using the significance analysis of microarrays (SAM) [91]. A list of genes with associated *q*-values [92] was generated using defined false discovery rates (FDRs) (we used 5% and 10%). The *q*-value gives the minimum value at which that gene will be considered significant. The cellular metabolomics dataset was analyzed by absolute quantification: a separate standard curve was completed for each metabolite, which allowed us to regress the density (area under the curve) to a known concentration of the metabolite. The standards curve was estimated by regressing density on concentration to obtain the linear coefficient. This coefficient, estimated independently for each metabolite, was then used to convert observed densities in the experimental data to quantities (µmoles) of the cellular metabolites.

The quantified data were then transformed by logs and analyzed by SAS Proc Mixed as a split plot with treatments; biological replicates within treatments were analyzed as whole plot effects, and cellular metabolites and cellular metabolite×treatment interactions were analyzed as split plot effects since all cellular metabolites sampled were correlated within a replicate. Replicates within treatments were the error term used to test treatment effects, while residue was used to test cellular metabolite×treatment interactions. The cellular metabolite×treatment interaction was the term of greatest interest, as it indicated which cellular metabolites were being affected by treatments. If the interaction was significant, means by cellular metabolite were compared for treatment effects by comparisons using single degrees of freedom.

For the toxicology experiments involving sugar feeding (Table S7) and mutant screening (Table S8), data were analyzed using the PROBIT procedure of SAS. The PROBIT procedure computes maximum likelihood estimates of the parameters of the probit equation using a modified Newton-Raphson algorithm. When the response Y is binary, with values 0 and 1, the probit equation is p = Pr(Y = 0) = C+(1−C)F(x'B), where B is a vector of parameter estimates, F is a cumulative distribution function (normal, logistic, or extreme value), x is a vector of explanatory variables, p is the probability of a response, C is the natural (threshold) response rate. The PROBIT procedure fits a common slope cumulative model, which is a parallel-line regression model based on the cumulative probabilities of the response categories rather than on their individual probabilities. The cumulative model has the form Pr (Y<i|x) = F(α+x'B). In our case we used the cumulative normal distribution for F, and x is log(time). The LT_50_ is estimated as LT_50_ = −α/B because when Pr (Y<i|x) = .5, the x corresponds to the center of the standardized normal distribution, or when α+x'B = 0. We estimated the LT_50_ with at least 5 independent data sets for each mutant. The error variance for the LT_50_ was estimated empirically as the variation among replicate estimates of the LT_50_ for that mutant and pooled across mutants. The combined data set was then analyzed by Proc GLM with mutants as treatments. Because there were different numbers of replicates per treatment, least squares means were computed for each mutant with corresponding standard error of the mean. Means were separated using planned comparison to the control only to control experiment wise error rate.

### DNA transcription factor binding motif analyses

The method of analysis was as described by Li *et al.* [93]. Transcription factor binding motifs (TFBMs) may regulate gene transcription in response to METH. Thus, we assessed the potential TFBMs of the 18 up-regulated genes (5% FDR) and 5 down-regulated genes (10% FDR) in response to METH treatment. The promoter regions near the genes were analyzed. The analysis included the 800 bp upstream and 200 bp downstream region from the transcription start site (TSS) of the gene. The distance indicates a dissimilarity measurement between any pair of position weight matrices (PWMs), so the smaller the value, the more similarity between the PWM and the mammalian TFBM. Distances of 0.1 or less indicated that the *D. melanogaster* TFBM was very similar to the respective mammalian one.

### Proteomic analyses

#### Sample preparation

Proteins were denatured and reduced with 8 M Urea (Fischer Scientific) and 10 mM dithiothreitol (DTT) (Fischer Scientific) for 1.5 hours at 37°C followed by further reduction and alkylation with 0.5% Triethylphosphine (TEP), 2% 2-Iodoethanol and 97.5% Acetonitrile for 1.5 hours at 37°C. Proteins were trypsin digested at a ratio of 1∶50 (w/w trypsin/protein) overnight at 37°C. The supernatant was removed and applied to a C18 microspin column (Nest Group, Southborough, MA) for buffer exchange and desalting. The resulting peptides were dried down and resuspended in 100 µl 0.01% TFA in water.

#### NanoLC-Chip-MS

The peptides (0.5 µg) were concentrated on the on-chip 300SB-C18 enrichment column at a flow rate of 4 µl/min for 5 minutes and separated with the on-chip C-18 reversed phase ZORBAX 300SB-C18 (0.75 µm×150 mm; Agilent) analytical column coupled to the electrospray ionization (ESI) source of the ion trap mass spectrometer (1100 Series LC equipped with HPLC chip interface, Agilent, XCT Plus, Agilent). A 55 min linear gradient from 5%–35% buffer B (100% acetonitrile, 0.01% TFA) at a rate of 300 nl/min, followed by a 10 minute gradient from 35%–100% buffer B was used to elute the column. After elution of the column, an isocratic flow (5% buffer B) at 300 nl/min was used for equilibration.

#### NanoLC-Chip-MS/MS and targeted MS/MS

Peptides were separated on a nanoLC-Chip system (1100 Series LC equipped with HPLC chip interface, Agilent, Santa Clara, CA) using the same platform as described above. Automated MS/MS spectra were acquired during the run in the data-dependent acquisition mode with the selection of the three most abundant precursor ions.

#### Data mining

The raw data files were converted into mzXML format using Bruker's CompassXport program and then analyzed using the “Proteomics Discovery Pipeline” (PDP). A chi-square statistical analysis was used to determine the significant peaks that were present in one group (*e.g.* treated) but not in the other (untreated). Peaks present in both sample groups but with significantly different intensities were evaluated by the standard two-sample t-test. The peptide peaks were ranked by their *p*-values and by their fold-change. The cut-off values were set at a 5% false discovery rate (*p*-value<0.05) and 2-fold or greater change in protein quantity. All peaks with *p*-values less than the cut-off were selected as differentially expressed peptides between the treated and untreated groups.

#### Protein identification

NanoLC-Chip-MS/MS results were analyzed using Spectrum Mill A.03.02.060 software (Agilent Technologies) and searches were performed against the National Institutes of Health National Center for Biotechnology Information (NCBI) protein database specifically for *Drosophila*. The parameters of the search were as follows: no more than two tryptic miscleavages allowed, cysteine searched as iodoethanol, 1.0 Da peptide mass tolerance and 0.7 Da MS/MS mass tolerance. Only peptides with a score of 6 or higher and %SPI of 60 or higher were considered true positives.

#### Overlap of MS and MS/MS data

A significant peak list, a treated peptide/protein list and an untreated peptide/protein list were generated from MS and MS/MS data, and the lists were combined. The MS and MS/MS raw data were compared to guarantee that the molecular information [m/z (+/−0.7 Da), retention time (+/−0.5 min), charge state] and chromatographic patterns were the same.

#### Gene ontology and KEGG analyses

The GeneChip *Drosophila* Genome 2.0 Array contains probe sets interrogating 18,952 genes from *Drosophila*, 14,705 of which used in the design of this array can be found in Flybase (http://flybase.org/). A combination of fold-change and test *p*-value methods are used to identify differentially expressed genes between control and METH treatments. For this method, genes were ordered on *p*-values derived from the t-statistic and reported only when a fold-change was greater than the given threshold–a practice commonly used in cDNA microarray data analysis [94]. Cutoff was set by a *p* value≤0.005 and the absolute fold-change ≥1.5 to choose differentially expressed genes.

Gene ontology categorization and pathway comparison were performed using the following databases: gene ontology (GO) system (http://www.geneontology.org) [95] and Kyoto Encyclopedia of Genes and Genomes (http://www.genome.ad.jp/kegg/) [96]. Fisher's exact test was used to test the statistical significance of associations between the gene list and expression changes and function set [97]. Significance levels for pathway comparisons were set by hit number >2, allowing any assumptions about the shape of sampling distribution of population to be avoided.

#### Network analyses

Differentially expressed transcripts and proteins identified in this study were compared with known networks in MetaCore integrated knowledge database using statistical tests and scoring for network relevance to the dataset, functional processes, cellular pathways and transcription factors of GeneGo software (http://www.genego.com/metacore.php) and DAVID. MetaCore™ is based on a proprietary manually curated database of human protein-protein, protein-DNA and protein compound interactions, metabolic and signaling pathways for human, mouse and rat, supported by proprietary ontologies and controlled vocabulary. The most highly significant pathways are summarized in Figure S2 and S3.

## Supporting Information

Figure S1 **Human gene orthologs of proteins in *Drosophila melanogaster* associated with the METH response.** *Drosophila* genes and their respective human gene orthologs were compared using David annotation software. The genes found to have the same function in *Drosophila* and humans were used to create the pie chart. The genes (Entrez_GeneID) observed for each of the pathways are: glycolysis (31532, 33351, 33824, 35728, 42185, 42620, and 43447), biotin metabolism (31551, 32095), steroid metabolism (53507 and 53511), oxidative phosphorylation (42591, 43829, 37617, 42291, and 41550), pyruvate metabolism (42620 and 42185), lipid metabolism (33824 and 33839), amino acid metabolism (41561), apoptosis and survival (35748), citrate cycle (42185), and starch and sucrose metabolism (53507 and 326264).
(TIF)

Figure S2 **Regulatory process maps based on proteomic and transcriptomic data.** Statistically significant regulatory process maps (networks) using genomic and proteomic data from *Drosophila melanogaster* treated with METH. Microarray and protein chip data are shown in red and blue, respectively. The networks maps were identified using the MetaCore integrated knowledge database.
(TIF)

Figure S3 **Regulatory metabolic maps based on proteomic and transcriptomic data.** Statistically significant metabolic maps (networks) in *Drosophila melanogaster* associated METH treatment, based on proteomic and transcriptomic data. Microarray and protein chip data are shown in red and blue, respectively. Common pathways are given in green. The network maps were identified using the MetaCore integrated knowledge database.
(TIF)

Figure S4 **Transcriptional factor binding motifs (TFBMs) impacted by METH exposure.** (A) TFBMs detected from 17 of up-regulated genes with <5% FDR. (B) TFBMs detected from 5 of down-regulated genes with <10% FDR. Sequence logo was generated using the WEBLOGO program.
(TIF)

Figure S5 **Transcriptional factor binding motifs (TFBMs) associated with over-transcribed genes.** Over-transcribed transcripts by methamphetamine in *Drosophila melanogaster* and the possible transcription factor binding motifs (TFBMs) relative to the gene transcription start site (TSS). Different symbols represent possible motifs. All transcripts are labeled with their respective gene names.
(TIF)

Figure S6 **Transcriptional factor binding motifs (TFBMs) associated with under-transcribed genes.** Under-transcribed genes in *Drosophila melanogaster* in response to treatment with methamphetamines and the possible transcription factor binding motifs (TFBMs) relative to the gene transcription start site (TSS). Different symbols represent different possible motifs. All transcripts are labeled with their respective gene names.
(TIF)

Figure S7 **Trehalose levels of METH-fed insects monitored by gas chromatography/mass spectrometry (GC/MS).** (A) GC/MS chromatogram of trehalose (the *x*-axis represents the retention time); the dotted line represents the control sample; the continuous line represents the METH-fed sample at mass 73. (B) Log scale of the area of control vs. METH with standard error bars (*P*<0.01). (C) Spectrum of trehalose. (D) Structure of trehalose that had been silylated using N-Methyl-N-trifluoroacetamide, Sialylation reagent (MSTFA) reagent.
(TIF)

Table S1 **Pathways are ranked by the number of proteins represented on protein chips and the number of genes represented on microarray.**
(XLS)

Table S2 **Proteins observed over- and under-expressed in methamphetamine-fed w1118** ***Drosophila melanogaster*** **adults.**
(XLS)

Table S3 ***Drosophila melanogaster*** **genes and human gene orthologs were compared using David annotation software.**
(XLS)

Table S4 **Potential transcription factor binding motifs (TFBMs) observed from over- and under-transcribed genes in w1118** ***Drosophila melanogaster*** **adults treated with methamphetamine.**
(XLS)

Table S5 **Genes that were differentially expressed in the microarray experiments, based on FDR analysis, at the q<10% levels in** ***Drosophila melanogaster*** **after 5-day-old flies were exposed to a diet containing 0.6% methamphetamine, as compared with flies reared on control diet.** Members of this gene set were used to predict potential transcription factor binding motifs.
(XLS)

Table S6 **The lethal time 50 (LT50) of** ***Drosophila*** **mutants fed media containing methamphetamine.**
(XLS)

Table S7 **The reverse and forward primers used for the qRT-PCR experiments.**
(XLS)

Table S8 **Genes that were differentially transcribed (based on qRT–PCR) in w1118** ***Drosophila melanogaster*** **adults after 5-day-old virgin male flies were exposed to a diet containing 0.6% methamphetamine, as compared with flies reared on control diet.**
(XLS)

Text S1 **Supplemental Results.**
(DOC)
