# Supplementary figures and images for: Systems-Scale Analysis Reveals Pathways Involved in Cellular Response to Methamphetamine
Source: PLoS One. 2011 Apr 20;6(4):e18215. doi: 10.1371/journal.pone.0018215 (PMC3080363; doi:10.1371/journal.pone.0018215)

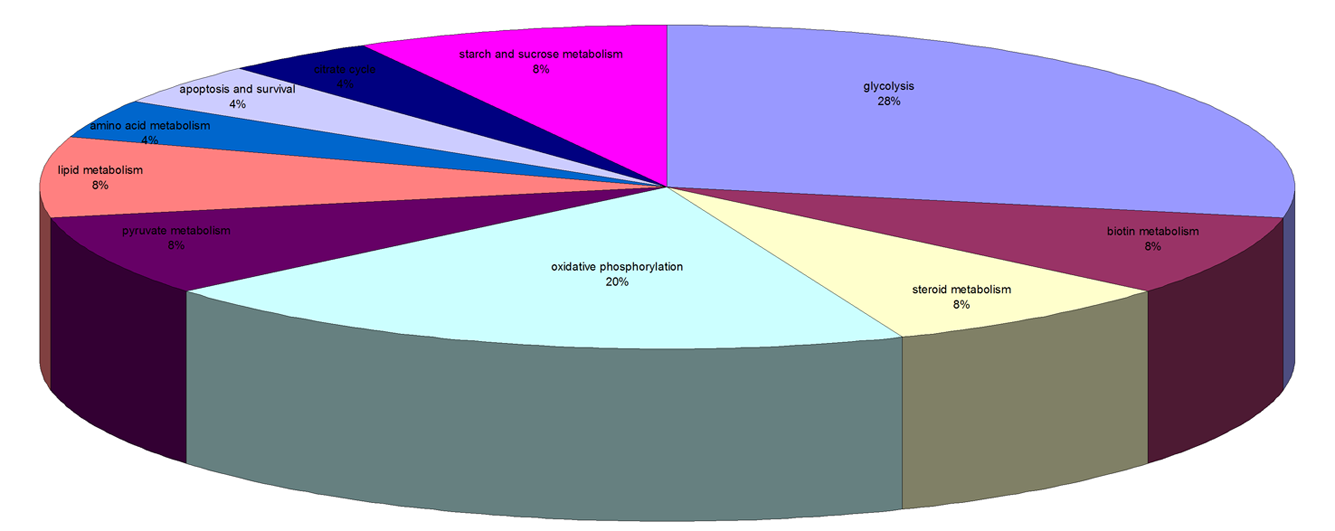

Supplement: Figure S1 — Human gene orthologs of proteins in Drosophila melanogaster associated with the METH response. Drosophila genes and their respective human gene orthologs were compared using David annotation software. The genes found to have the same function in Drosophila and humans were used to create the pie chart. The genes (Entrez_GeneID) observed for each of the pathways are: glycolysis (31532, 33351, 33824, 35728, 42185, 42620, and 43447), biotin metabolism (31551, 32095), steroid metabolism (53507 and 53511), oxidative phosphorylation (42591, 43829, 37617, 42291, and 41550), pyruvate metabolism (42620 and 42185), lipid metabolism (33824 and 33839), amino acid metabolism (41561), apoptosis and survival (35748), citrate cycle (42185), and starch and sucrose metabolism (53507 and 326264). (TIF) [file pone.0018215.s001.tif]

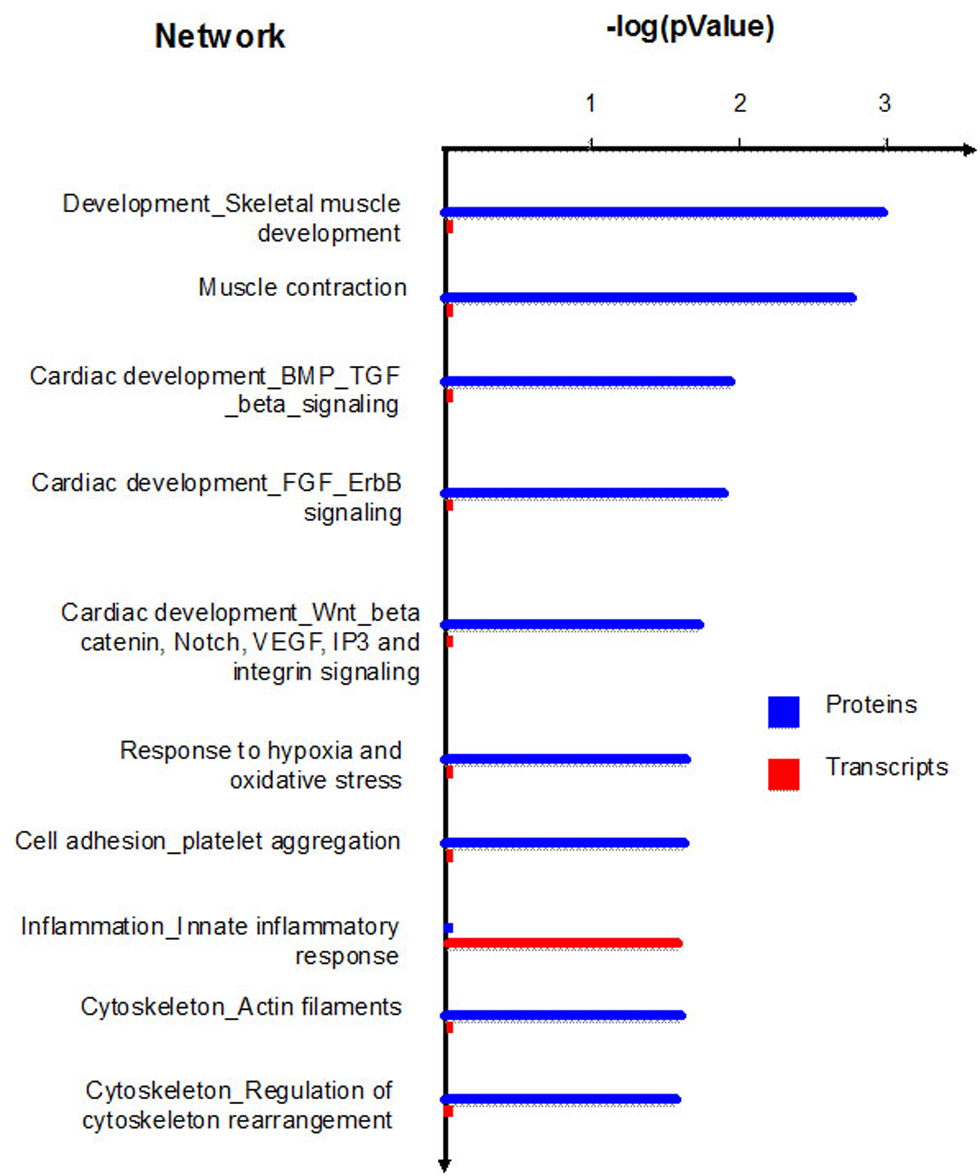

Supplement: Figure S2 — Regulatory process maps based on proteomic and transcriptomic data. Statistically significant regulatory process maps (networks) using genomic and proteomic data from Drosophila melanogaster treated with METH. Microarray and protein chip data are shown in red and blue, respectively. The networks maps were identified using the MetaCore integrated knowledge database. (TIF) [file pone.0018215.s002.tif]

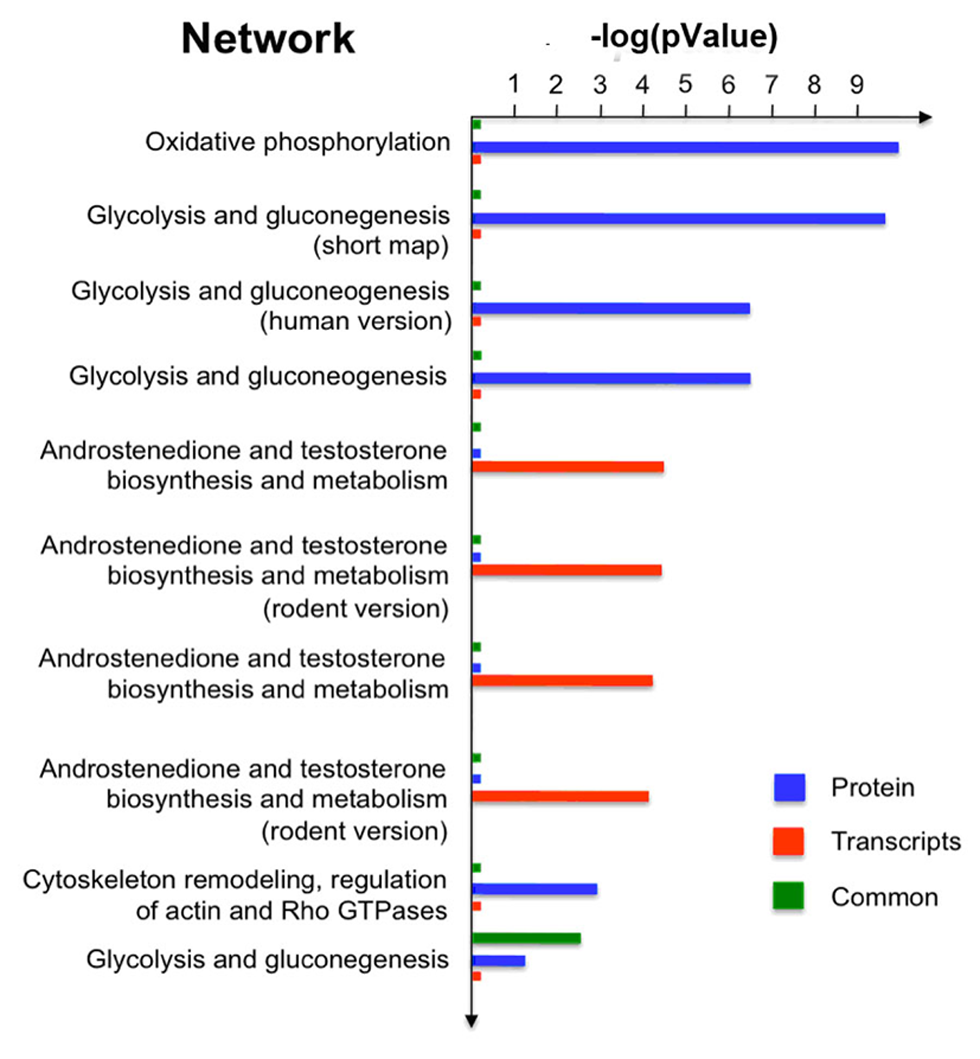

Supplement: Figure S3 — Regulatory metabolic maps based on proteomic and transcriptomic data. Statistically significant metabolic maps (networks) in Drosophila melanogaster associated METH treatment, based on proteomic and transcriptomic data. Microarray and protein chip data are shown in red and blue, respectively. Common pathways are given in green. The network maps were identified using the MetaCore integrated knowledge database. (TIF) [file pone.0018215.s003.tif]

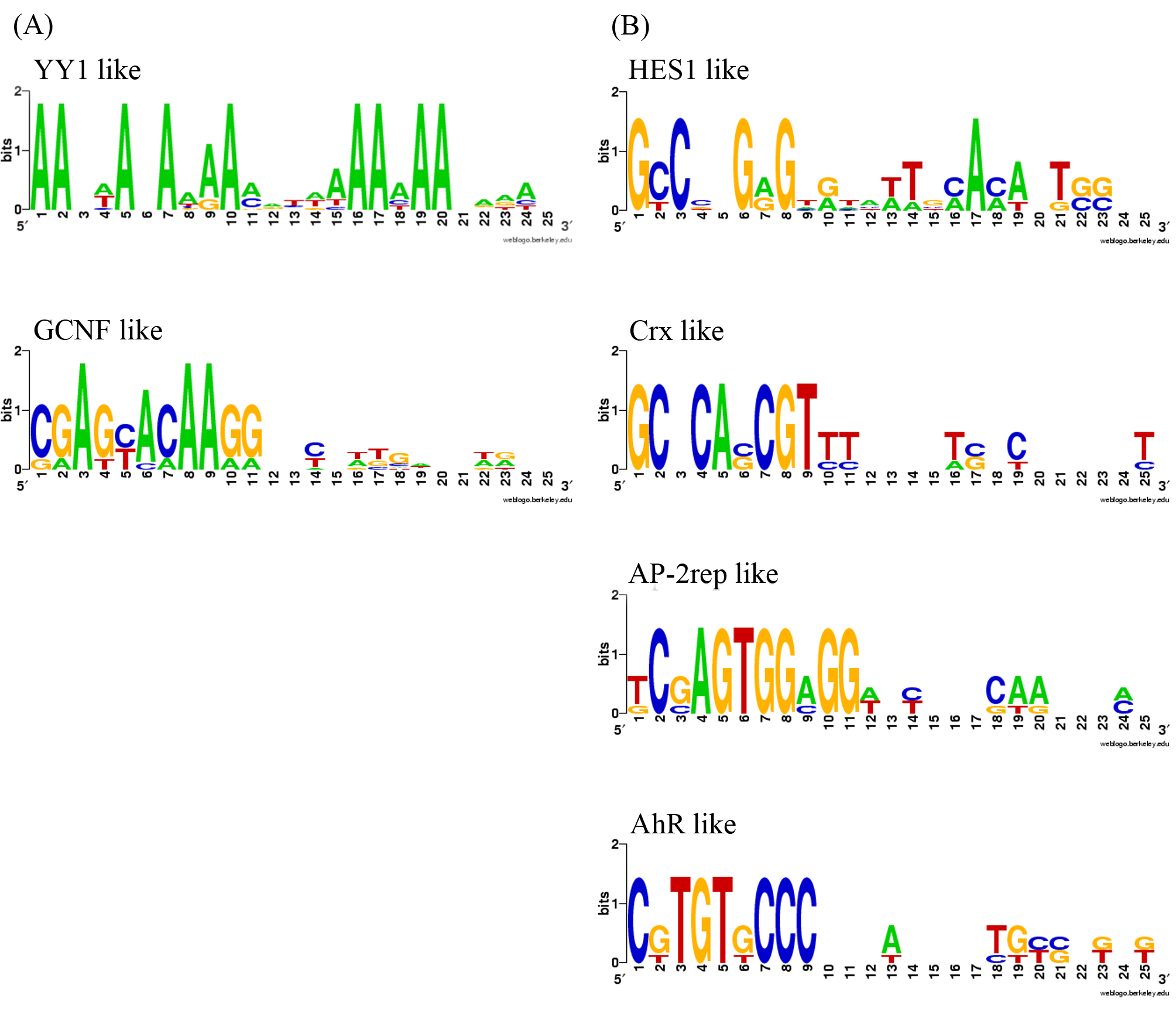

Supplement: Figure S4 — Transcriptional factor binding motifs (TFBMs) impacted by METH exposure. (A) TFBMs detected from 17 of up-regulated genes with <5% FDR. (B) TFBMs detected from 5 of down-regulated genes with <10% FDR. Sequence logo was generated using the WEBLOGO program. (TIF) [file pone.0018215.s004.tif]

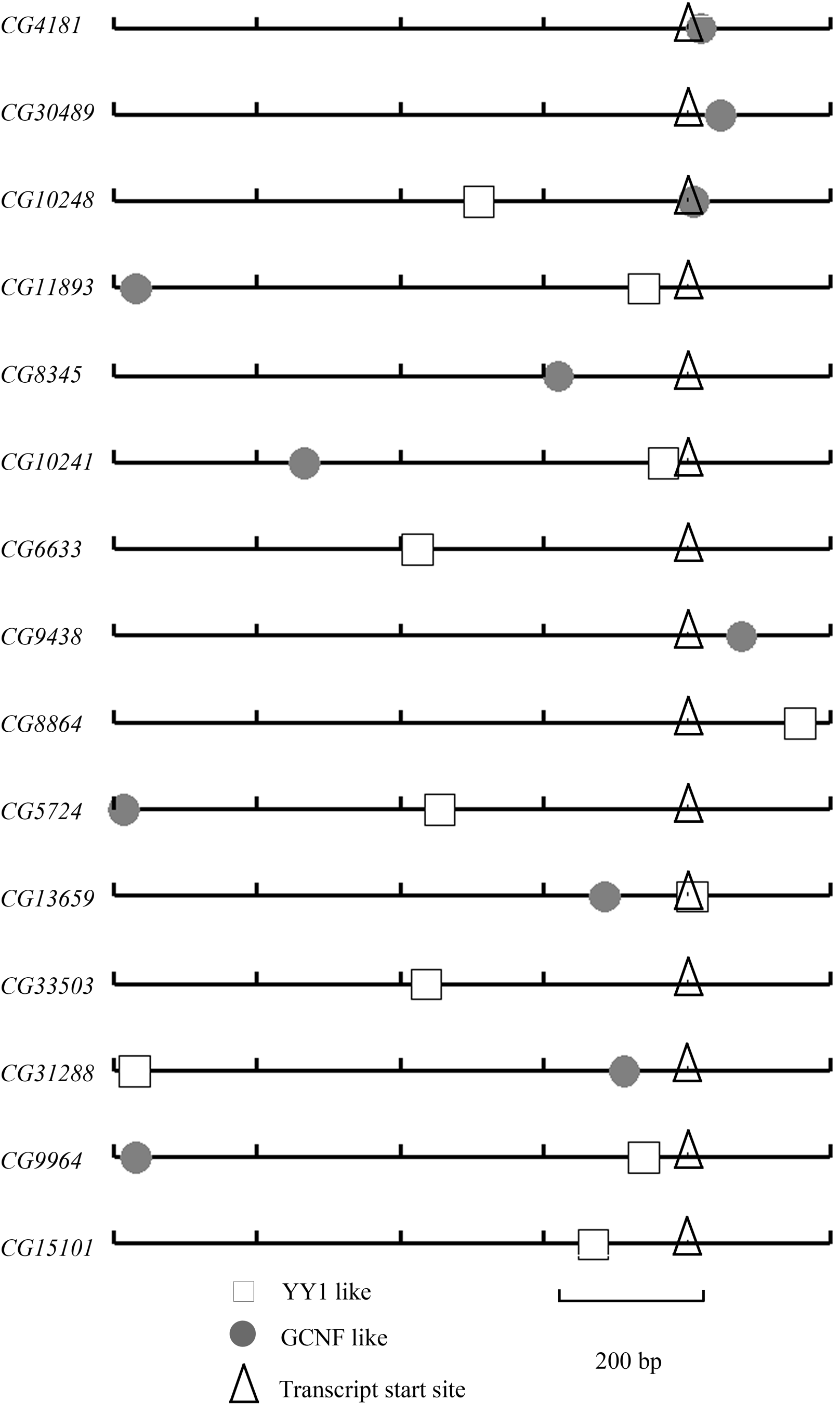

Supplement: Figure S5 — Transcriptional factor binding motifs (TFBMs) associated with over-transcribed genes. Over-transcribed transcripts by methamphetamine in Drosophila melanogaster and the possible transcription factor binding motifs (TFBMs) relative to the gene transcription start site (TSS). Different symbols represent possible motifs. All transcripts are labeled with their respective gene names. (TIF) [file pone.0018215.s005.tif]

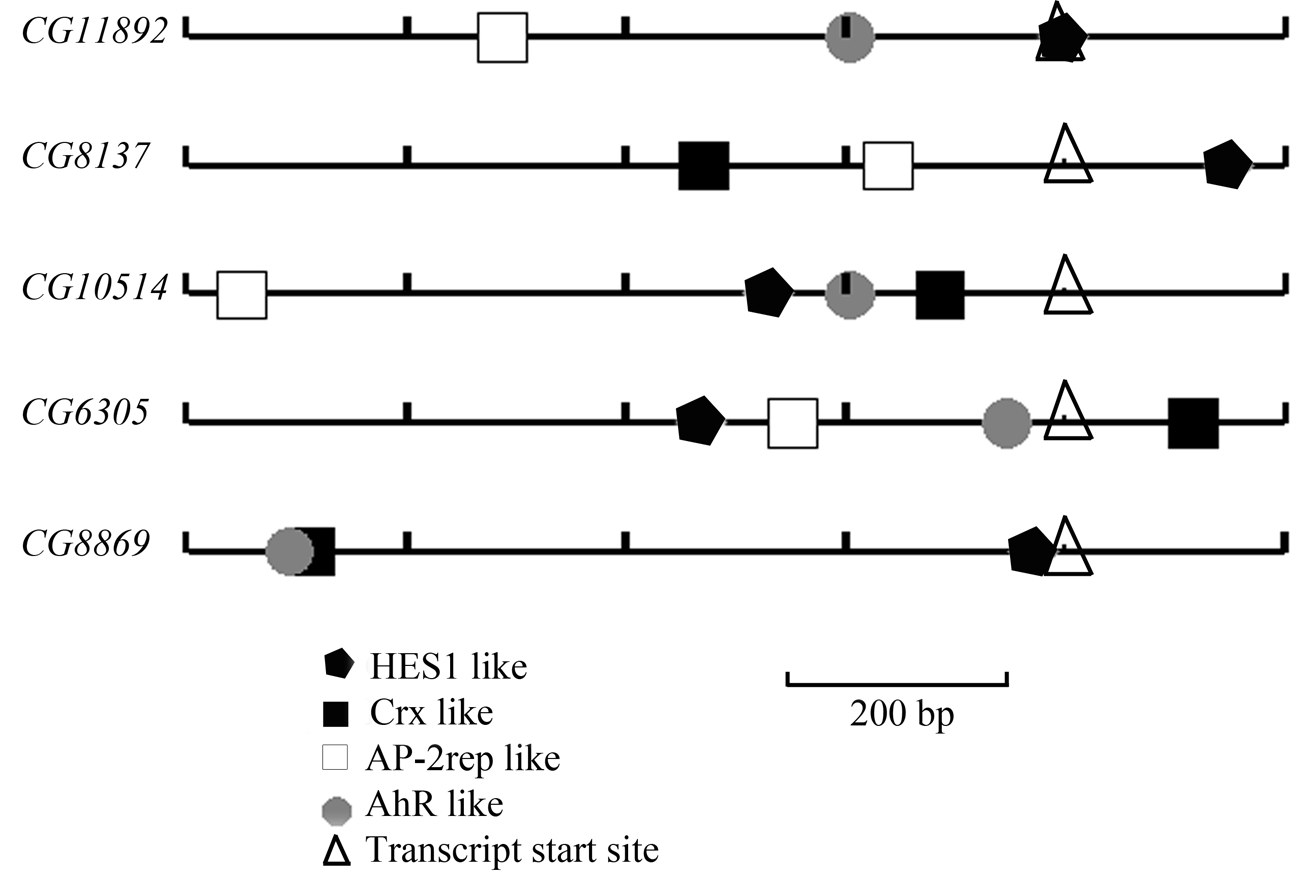

Supplement: Figure S6 — Transcriptional factor binding motifs (TFBMs) associated with under-transcribed genes. Under-transcribed genes in Drosophila melanogaster in response to treatment with methamphetamines and the possible transcription factor binding motifs (TFBMs) relative to the gene transcription start site (TSS). Different symbols represent different possible motifs. All transcripts are labeled with their respective gene names. (TIF) [file pone.0018215.s006.tif]

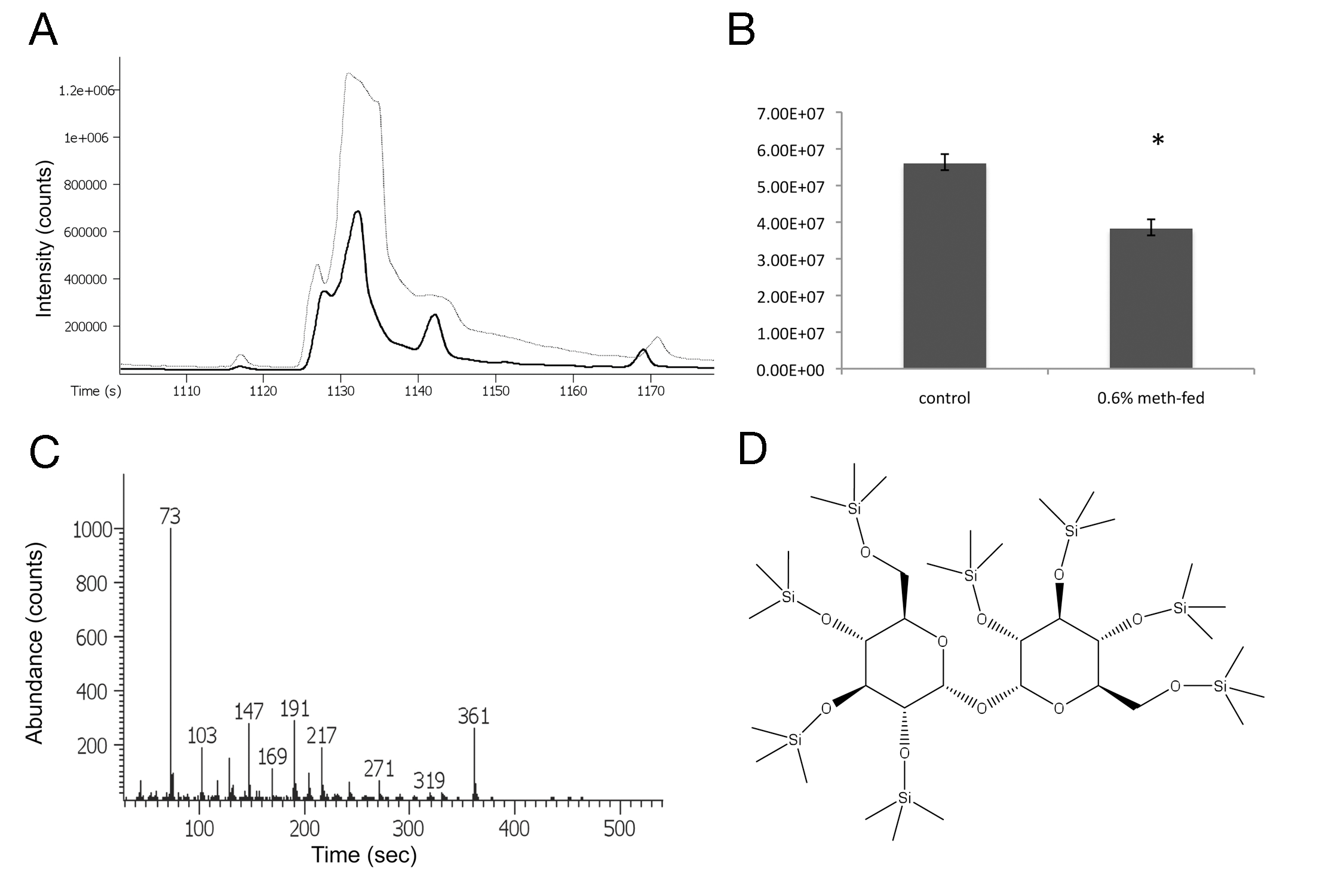

Supplement: Figure S7 — Trehalose levels of METH-fed insects monitored by gas chromatography/mass spectrometry (GC/MS). (A) GC/MS chromatogram of trehalose (the x-axis represents the retention time); the dotted line represents the control sample; the continuous line represents the METH-fed sample at mass 73. (B) Log scale of the area of control vs. METH with standard error bars (P<0.01). (C) Spectrum of trehalose. (D) Structure of trehalose that had been silylated using N-Methyl-N-trifluoroacetamide, Sialylation reagent (MSTFA) reagent. (TIF) [file pone.0018215.s007.tif]
