# Supplementary material for: Systems-Scale Analysis Reveals Pathways Involved in Cellular Response to Methamphetamine
Source: PLoS One. 2011 Apr 20;6(4):e18215. doi: 10.1371/journal.pone.0018215 (PMC3080363; doi:10.1371/journal.pone.0018215)
Supplement: Text S1 — Supplemental Results. (DOC) [file pone.0018215.s016.doc]

**Supplementary Online Materials**

Supplemental Results

**Potential transcription factor binding motifs (TFBMs) associated with the METH response**

Similar to Dieterich *et al*. [1], Song *et al*. [2] and Li *et al*. [3,4], we employed a false discovery rate (FDR) analysis of microarray data followed by qRT-PCR (Tables S4, S5, S7, S8) in order to determine candidate genes for identifying potential transcription factor binding motifs (TFBMs) associated with xenobiotic responses. We added “-like” to possible TFBMs we observed from *Drosophila,* since the patterns of these potential TFBMs were similar to those of mammals having the corresponding functions (Figure S4-6).

Of the potential TFBMs, Yin Yang 1-like (YY1-like) was the most similar to its mammalian counterpart (Table S4). Previously linked with the METH response in mice [5], YY1 is a 65 kDa zinc finger transcription factor which appears to be highly conserved across vertebrates [6,7]. YY1 is associated with histone acetyltransferase and histone deacetylase cofactors. Amphetamines are known to increase histone H4 acetylation in the striatum of mice [8], with chronic use increasing **histone** H3 methylation on the c-*fos* promoter [9]. Additionally, the YY1 response element, the c-Myc promoter, and α-enolase play an interactive role in tumorigenesis [10]. YY1 is (i) associated with increases in human heart failure (a component of the METH syndrome in humans) and (ii) a negative regulator of the alpha myosin heavy chain (MyHC) [11], a protein responsive to METH treatment both in mammals [12] and in *Drosophila* (Table S2).

**Proteases**

From the proteomic data, we also observed a 10-fold increase in arginine kinase, a 10-fold decrease in protein kinase D, and a 5-fold decrease in serine protease inhibitor 2. Oxidative stress has been previously reported to induce arginine kinase expression [13] and protein kinase D activation in intact cells in a dose- and time-dependent manner [14]. Protein kinases have also been linked to the pathogenesis of Parkinson's disease [15] and to responses in the brain to METH treatment [16,17]. Trypsin and trypsin-like serine proteases and their inhibitors are known to play very important roles in brain-related (i) neural development and plasticity, and (ii) neuroregeneration and neurodegeneration [18,19,20,21,22,23,24,25,26]. However, serine proteases and several other proteases increase oxidative stress, indicating the potential for direct interaction between proteases and reactive oxygen species (ROS) that contributes to the overall enhancement of oxidative stress that leads to cell death [27].

**Cytochrome P450s and glutathione-S transferases**

Our microarray analyses (using KEGG and FDR) demonstrated that cytochrome P450 and glutathione S transferase (GST) transcripts were up-regulated in response to METH (Table S1, S5). METH is known to induce P450 expression and be metabolized by cytochrome P450s in rats [28], results that are consistent with the up-regulation of P450s detected in *Drosophila*. However, the toxic actions of some chemicals (*e.g.* 2,3,7,8-tetrachlorodibenzo-*p*-dioxin) have been associated with the ROS produced by the induction of P450s [29,30]. Different drugs selectively induce P450s, and P450 families 1, 2, 3, and 4 have been reported to play a role in the generation of ROS [31,32]. Seven GST genes were up-regulated in response to METH in *Drosophila*; GSTs can be induced in the kidney and hypothalamus of rats by d-amphetamine treatment [33,34]. In addition to detoxifying drugs, GSTs also allow organisms to reduce oxidative stress [35,36]. The induced GSTs in METH-treated flies may be involved in drug detoxification, a response to oxidative stress.

In addition to their roles in detoxifying xenobiotics and responding to oxidative stress, several cytochrome P450 and GSTs in *Drosophila* may also affect spermatogenesis. *Cyp6a2,* which is up-regulated in flies challenged with METH, is expressed in the testes, vas deferens, ejaculatory duct, and the sperm pump [37]. *GstE1* was up-regulated in METH-treated flies and has been associated with the gene encoding farnesyl pyrophosphatase synthetase*,* an enzyme in the testes of rats that is essential for the migration of germ cells during spermatogenesis and for the synthesis of cholesterol [38]. The cholesterol/phospholipid ratio of the sperm plasma membrane determines whether the sperm can successfully penetrate an ovum [39]. In *Drosophila*, farnesyl pyrophosphate is converted into juvenile hormone (JH) [40]. Deficiencies in JH have been implicated in sterility and aberrant sexual behavior [41].

**Oxidative stress**

METH and its analogs have been associated with both oxidative stress and heart disease [42,43,44]. Dynein, a motor protein that converts the chemical energy contained in ATP into the mechanical energy of movement, is prone to oxidation and dimerization [45]. Under oxidative stress, tropomyosin modification is associated with heart disease due to cytoskeletal remodeling and myocardial dysfunction [46]. In addition, the tyrosine nitration of MyHCs alters the structure of cardiac myocytes [47]. It remains to be determined if METH treatment causes physical damage to *Drosophila*’s dorsal tube, the insect equivalent of the heart. If it does, the METH-*Drosophila* model could provide insights into evolutionarily conserved aspects of human heart failure [48].

METH is a weak base capable of causing the alkalinization of acidic vesicles and organelles of cultured midbrain dopamine (DA) neurons, inhibiting dopamine compartmentalization [49]. Gnegy *et al.* demonstrated that weak bases cause the efflux of DA from acidic vesicles [50]. METH-induced toxicity is, in part, related to the increased cytosolic DA, which is rapidly metabolized, leading to the production of ROS [51,52].

**Ferritin**

Iron chelators, such ferritin, are multifunctional proteins that are involved in several metabolic pathways including Fe homeostasis. METH treatment is known to cause ferritin increases in the *substantia nigra pars reticulata* and *globus pallidus* of vervet monkeys [53]. These are parts of the brain that are involved in reward, addiction, and movement, and pre-filtering external stimuli, respectively. Young vervet monkeys treated with METH had ferritin levels comparable to those of much older drug-naïve monkeys. The negative consequences of chronic ferritin H-chain over-expression have been associated with aging in mice [54]. Increased brain iron and ferritin are known to occur in the *substantia nigra zona compacta* of Parkinson's patients [55]; long-term METH use can result in Parkinson's-like symptoms. Conversely, protection from oxidative stress has been shown in young mice that over-express the ferritin H-chain, because of the iron-chelating properties of ferritin [56,57]. In contrast to the observations in mammalian systems [53], we observed a 10-fold decrease in ferritin 1 heavy chain homologue protein in METH-treated flies (Table S2). It remains to be determined if this change in ferritin expression is associated with the brain in *Drosophila*.

**Steroidal systems**

The analysis of self-regulation through transcription factors also revealed the differential expression of genes and proteins known in mammals to be associated with androgen receptors, c-Myc, and p53 (Figure 3). In mammals, amphetamines are known to influence testosterone levels and the production of progesterone by increasing the activity of specific P450s [58]. In mice, METH causes an up-regulation of c-Myc, which is involved in apoptosis, both at mRNA and protein levels [59]. The tumor suppressor p53 is associated with the long-term neurotoxic effects of METH [60]. So, unsurprisingly, p53 knockout mice had fewer side-effects due to METH treatment than did control mice. METH-treated wild-type mice had reduced dopamine transporter mRNA and numbers of tyrosine hydroxylase-positive cells in their *substantia nigra pars compacta* and the ventral tegmental area; homozygous p53 knockout mice were not affected by METH treatment.

­­­­References

1. Dieterich C, Rahmann S, Vingron M (2004) Functional inference from non-random distributions of conserved predicted transcription factor binding sites. Bioinformatics 20 Suppl 1: i109-115.

2. Song J, Bjarnason J, Surette MG (2005) The identification of functional motifs in temporal gene expression analysis. Evol Bioinform Online 1: 84-96.

3. Li HM, Buczkowski G, Mittapalli O, Xie J, Wu J, et al. (2008) Transcriptomic profiles of *Drosophila melanogaster* third instar larval midgut and responses to oxidative stress. Insect Mol Biol 17: 325-339.

4. Li HM, Sun L, Mittapalli O, Muir WM, Xie J, et al. (2009) Bowman-Birk inhibitor affects pathways associated with energy metabolism in *Drosophila melanogaster*. Insect Mol Biol. 19: 303-313.

5. Ryu NK, Yang MH, Jung MS, Jeon JO, Kim KW, et al. (2007) Gene expression profiling of rewarding effect in methamphetamine treated Bax-deficient mouse. J Biochem Mol Biol 40: 475-485.

6. Shi Y, Lee JS, Galvin KM (1997) Everything you have ever wanted to know about Yin Yang 1. Biochim Biophys Acta 1332: F49-66.

7. Shi Y, Seto E, Chang LS, Shenk T (1991) Transcriptional repression by YY1, a human GLI-Kruppel-related protein, and relief of repression by adenovirus E1A protein. Cell 67: 377-388.

8. Kalda A, Heidmets LT, Shen HY, Zharkovsky A, Chen JF (2007) Histone deacetylase inhibitors modulates the induction and expression of amphetamine-induced behavioral sensitization partially through an associated learning of the environment in mice. Behav Brain Res 181: 76-84.

9. Renthal W, Carle TL, Maze I, Covington HE, 3rd, Truong HT, et al. (2008) Delta FosB mediates epigenetic desensitization of the c-fos gene after chronic amphetamine exposure. J Neurosci 28: 7344-7349.

10. Hsu K-W, Hsieh R-H, Lee Y-HW, Chao C-H, Wu K-J, et al. (2008) The activated Notch1 receptor cooperates with {alpha}-Enolase and MBP-1 in modulating c-myc activity. Mol Cell Biol 28: 4829-4842.

11. Sucharov CC, Dockstader K, McKinsey TA (2008) YY1 protects cardiac myocytes from pathologic hypertrophy by interacting with HDAC5. Mol Biol Cell 19: 4141-4153.

12. Inoue H, Nakatome M, Terada M, Mizuno M, Ono R, et al. (2004) Maternal methamphetamine administration during pregnancy influences on fetal rat heart development. [corrected]. Life Sci 74: 1529-1540.

13. Miranda MR, Canepa GE, Bouvier LA, Pereira CA (2006) Trypanosoma cruzi: Oxidative stress induces arginine kinase expression. Exp Parasitol 114: 341-344.

14. Waldron RT, Rozengurt E (2000) Oxidative stress induces protein kinase D activation in intact cells. Involvement of Src and dependence on protein kinase C. J Biol Chem 275: 17114-17121.

15. Shen J (2004) Protein kinases linked to the pathogenesis of Parkinson's disease. Neuron 44: 575-577.

16. Suemaru J, Akiyama K, Tanabe Y, Kuroda S (2000) Methamphetamine decreases calcium-calmodulin dependent protein kinase II activity in discrete rat brain regions. Synapse 36: 155-166.

17. Uemura K, Aki T, Yamaguchi K, Yoshida K (2003) Protein kinase C-epsilon protects PC12 cells against methamphetamine-induced death: possible involvement of suppression of glutamate receptor. Life Sci 72: 1595-1607.

18. Shimizu C, Yoshida S, Shibata M, Kato K, Momota Y, et al. (1998) Characterization of recombinant and brain neuropsin, a plasticity-related serine protease. J Biol Chem 273: 11189-11196.

19. Molinari F, Rio M, Meskenaite V, Encha-Razavi F, Auge J, et al. (2002) Truncating neurotrypsin mutation in autosomal recessive nonsyndromic mental retardation. Science 298: 1779-1781.

20. Xi G, Reiser G, Keep RF (2003) The role of thrombin and thrombin receptors in ischemic, hemorrhagic and traumatic brain injury: deleterious or protective? J Neurochem 84: 3-9.

21. Matsumoto-Miyai K, Ninomiya A, Yamasaki H, Tamura H, Nakamura Y, et al. (2003) NMDA-dependent proteolysis of presynaptic adhesion molecule L1 in the hippocampus by neuropsin. J Neurosci 23: 7727-7736.

22. Terayama R, Bando Y, Yamada M, Yoshida S (2005) Involvement of neuropsin in the pathogenesis of experimental autoimmune encephalomyelitis. Glia 52: 108-118.

23. Sheehan JJ, Tsirka SE (2005) Fibrin-modifying serine proteases thrombin, tPA, and plasmin in ischemic stroke: a review. Glia 50: 340-350.

24. Numajiri T, Mitsui S, Hisa Y, Ishida T, Nishino K, et al. (2006) The expression of a motoneuron-specific serine protease, motopsin (PRSS12), after facial nerve axotomy in mice. J Plast Reconstr Aesthet Surg 59: 393-397.

25. Terayama R, Bando Y, Murakami K, Kato K, Kishibe M, et al. (2007) Neuropsin promotes oligodendrocyte death, demyelination and axonal degeneration after spinal cord injury. Neuroscience 148: 175-187.

26. Wang Y, Luo W, Reiser G (2008) Trypsin and trypsin-like proteases in the brain: proteolysis and cellular functions. Cell Mol Life Sci 65: 237-252.

27. Aoshiba K, Yasuda K, Yasui S, Tamaoki J, Nagai A (2001) Serine proteases increase oxidative stress in lung cells. Am J Physiol Lung Cell Mol Physiol 281: L556-564.

28. Dostalek M, Jurica J, Pistovcakova J, Hanesova M, Tomandl J, et al. (2007) Effect of methamphetamine on cytochrome P450 activity. Xenobiotica: The fate and safety evaluation of foreign compounds in biological systems 37: 1355 - 1366.

29. Park JY, Shigenaga MK, Ames BN (1996) Induction of cytochrome P4501A1 by 2,3,7,8-tetrachlorodibenzo-p-dioxin or indolo(3,2-b)carbazole is associated with oxidative DNA damage. Proc Natl Acad Sci U S A 93: 2322-2327.

30. Shertzer HG, Nebert DW, Puga A, Ary M, Sonntag D, et al. (1998) Dioxin causes a sustained oxidative stress response in the mouse. Biochem Biophys Res Commun 253: 44-48.

31. Caro AA, Cederbaum AI (2004) Oxidative stress, toxicology, and pharmacology of CYP2E1. Annu Rev Pharmacol Toxicol 44: 27-42.

32. Imaoka S, Osada M, Minamiyama Y, Yukimura T, Toyokuni S, et al. (2004) Role of phenobarbital-inducible cytochrome P450s as a source of active oxygen species in DNA-oxidation. Cancer Lett 203: 117-125.

33. Carvalho F, Fernandes E, Remiao F, de Lourdes Bastos M (1999) Effect of d-amphetamine repeated administration on rat antioxidant defences. Arch Toxicol 73: 83-89.

34. Carvalho F, Fernandes E, Remiao F, Gomes-Da-Silva J, Tavares MA, et al. (2001) Adaptative response of antioxidant enzymes in different areas of rat brain after repeated d-amphetamine administration. Addict Biol 6: 213-221.

35. Awasthi Y ZP, Awasthi S, Singhal S, Srivastava S, Piper J, Chaubey M, Petersen D, He N, Sharma R, Singh S, Khan M, Ansari G & Boor P. (1996) A new group of glutathione S-transferases with protective role against lipid peroxidation. In: Vermeulen NPE, Mulder GJ, Nieuwenhuyse H, Peters WHM, and van Bladeren PJ, editors. Glutathione S-transferases: Structure, Function and Clinical Implications. London: Taylor & Francis. pp. 111-124.

36. Sawicki R, Singh SP, Mondal AK, Benes H, Zimniak P (2003) Cloning, expression and biochemical characterization of one Epsilon-class (GST-3) and ten Delta-class (GST-1) glutathione S-transferases from *Drosophila melanogaster*, and identification of additional nine members of the Epsilon class. Biochem J 370: 661-669.

37. Giraudo M, Unnithan GC, Le Goff G, Feyereisen R (2010) Regulation of cytochrome P450 expression in *Drosophila*: Genomic insights. Pestic Biochem Physiol 97(2): 115-122.

38. Teruya JH, Salido EC, Edwards PA, Clarke CF (1991) Testis-specific transcripts of rat farnesyl pyrophosphate synthetase are developmentally regulated and localized to haploid germ cells. Biol Reprod 44: 663-671.

39. Cross NL (1998) Role of cholesterol in sperm capacitation. Biol Reprod 59: 7-11.

40. Cao L, Zhang P, Grant DF (2009) An insect farnesyl phosphatase homologous to the N-terminal domain of soluble epoxide hydrolase. Biochem Biophys Res Commun 380: 188.

41. Atalia S, Ruth W, Daniel S (1995) Genetic and molecular studies of apterous: A gene implicated in the juvenile hormone system of *Drosophila*. Archives of Insect Biochemistry and Physiology 30: 195-209.

42. Yu Q, Larson DF, Watson RR (2003) Heart disease, methamphetamine and AIDS. Life Sci 73: 129.

43. Watts DJ, McCollester L (2006) Methamphetamine-induced myocardial infarction with elevated troponin I. The American Journal of Emergency Medicine 24: 132.

44. Shenouda SK, Lord KC, McIlwain E, Lucchesi PA, Varner KJ (2008) Ecstasy produces left ventricular dysfunction and oxidative stress in rats. Cardiovasc Res 79: 662-670.

45. Gough NR (2008) Dynein Light Chain Connecting ROS to NF-{kappa}B. Sci Signal 1: ec309-.

46. Canton M, Skyschally A, Menabo R, Boengler K, Gres P, et al. (2006) Oxidative modification of tropomyosin and myocardial dysfunction following coronary microembolization. Eur Heart J 27: 875-881.

47. Hong SJ, Gokulrangan G, Schöneich C (2007) Proteomic analysis of age dependent nitration of rat cardiac proteins by solution isoelectric focusing coupled to nanoHPLC tandem mass spectrometry. Exp Gerontol 42: 639.

48. Wolf MJ, Rockman HA (2008) *Drosophila melanogaster* as a model system for genetics of postnatal cardiac function. Drug Discov Today Dis Models 5: 117-123.

49. Sulzer D, Rayport S (1990) Amphetamine and other psychostimulants reduce pH gradients in midbrain dopaminergic neurons and chromaffin granules: A mechanism of action. Neuron 5: 797.

50. Gnegy ME, Khoshbouei H, Berg KA, Javitch JA, Clarke WP, et al. (2004) Intracellular Ca2+ regulates amphetamine-induced dopamine efflux and currents mediated by the human dopamine transporter. Mol Pharmacol 66: 137-143.

51. Cadet JL, Brannock C (1998) Free radicals and the pathobiology of brain dopamine systems. Neurochem Int 32: 117.

52. Wrona MZ, Yang Z, Zhang F, Dryhurst G (1997) Potential new insights into the molecular mechanisms of methamphetamine-induced neurodegeneration. NIDA Res Monogr 173: 146-174.

53. Melega WP, Lacan G, Harvey DC, Way BM (2007) Methamphetamine increases basal ganglia iron to levels observed in aging. Neuroreport 18: 1741-1745.

54. Kaur D, Rajagopalan S, Chinta S, Kumar J, Di Monte D, et al. (2007) Chronic ferritin expression within murine dopaminergic midbrain neurons results in a progressive age-related neurodegeneration. Brain Res 1140: 188-194.

55. Jellinger K, Paulus W, Grundke-Iqbal I, Riederer P, Youdim MB (1990) Brain iron and ferritin in Parkinson's and Alzheimer's diseases. J Neural Transm Park Dis Dement Sect 2: 327-340.

56. Kaur D, Yantiri F, Rajagopalan S, Kumar J, Mo JQ, et al. (2003) Genetic or pharmacological iron chelation prevents MPTP-induced neurotoxicity in vivo: a novel therapy for Parkinson's disease. Neuron 37: 899-909.

57. Wilkinson Jt, Di X, Schonig K, Buss JL, Kock ND, et al. (2006) Tissue-specific expression of ferritin H regulates cellular iron homoeostasis in vivo. Biochem J 395: 501-507.

58. Chen LY, Huang YL, Liu MY, Leu SF, Huang BM (2003) Effects of amphetamine on steroidogenesis in MA-10 mouse Leydig tumor cells. Life Sci 72: 1983-1995.

59. Thiriet N, Jayanthi S, McCoy M, Ladenheim B, Lud Cadet J (2001) Methamphetamine increases expression of the apoptotic c-myc and L-myc genes in the mouse brain. Brain Res Mol Brain Res 90: 202-204.

60. Hirata H, Cadet JL (1997) p53-knockout mice are protected against the long-term effects of methamphetamine on dopaminergic terminals and cell bodies. J Neurochem 69: 780-790.

61. Zhang R, Kai T, Sugimoto Y, Kusakabe T, Takasaki Y, et al. (1995) *Drosophila* *melanogaster* Aldolase: Characterization of the Isozymes {alpha}, {beta}, and {gamma} Generated from a Single Gene. J Biochem 118: 183-188.

62. Jacobs H, Stratmann R, Lehner CF (1998) A screen for lethal mutations in the chromosomal region 59AB suggests that bellwether encodes the alpha subunit of the mitochondrial ATP synthase in *Drosophila melanogaster*. Mol Gen Genet 259: 383.

63. Parsch J, Meiklejohn CD, Hauschteck-Jungen E, Hunziker P, Hartl DL (2001) Molecular evolution of the ocnus and janus genes in the *Drosophila* *melanogaster* species subgroup. Mol Biol Evol 18: 801-811.

64. Yang WM, Yao YL, Sun JM, Davie JR, Seto E (1997) Isolation and characterization of cDNAs corresponding to an additional member of the human histone deacetylase gene family. J Biol Chem 272: 28001-28007.

65. Zechel C (2005) The germ cell nuclear factor (GCNF). Mol Reprod Dev 72: 550-556.

66. Furukawa T, Morrow EM, Cepko CL (1997) Crx, a novel otx-like homeobox gene, shows photoreceptor-specific expression and regulates photoreceptor differentiation. Cell 91: 531-541.

67. Imhof A, Schuierer M, Werner O, Moser M, Roth C, et al. (1999) Transcriptional regulation of the AP-2alpha promoter by BTEB-1 and AP-2rep, a novel wt-1/egr-related zinc finger repressor. Mol Cell Biol 19: 194-204.

68. Tijet N, Boutros PC, Moffat ID, Okey AB, Tuomisto J, et al. (2006) Aryl hydrocarbon receptor regulates distinct dioxin-dependent and dioxin-independent gene batteries. Mol Pharmacol 69: 140-153.

69. Israel DI, Whitlock JP, Jr. (1983) Induction of mRNA specific for cytochrome P1-450 in wild type and variant mouse hepatoma cells. J Biol Chem 258: 10390-10394.

70. Sun W, Margam VM, Sun L, Buczkowski G, Bennett GW, et al. (2006) Genome-wide analysis of phenobarbital-inducible genes in *Drosophila melanogaster*. Insect Mol Bio 15: 455.

71. Willoughby L, Chung H, Lumb C, Robin C, Batterham P, et al. (2006) A comparison of *Drosophila melanogaster* detoxification gene induction responses for six insecticides, caffeine and phenobarbital. Insect Biochem Mol Bio 36: 934.
